# Molecular modeling studies and *in vitro* screening of dihydrorugosaflavonoid and its derivatives against *Mycobacterium tuberculosis*†

**DOI:** 10.1039/c8ra00636a

**Published:** 2018-03-16

**Authors:** Ninad V. Puranik, Pratibha Srivastava, Sagar Swami, Amit Choudhari, Dhiman Sarkar

**Affiliations:** Bioprospecting Group, Agharkar Research Institute G. G. Agarkar Road Pune 411004 Maharashtra India ninadv_puranik@yahoo.co.in psrivastava@aripune.org; Organic Chemistry Division, National Chemical Laboratory Dr Homi Bhabha Road Pune-411008 Maharashtra India d.sarkar@ncl.res.in; Savitribai Phule Pune University Pune-411007 India

## Abstract

Novel drug regimens against tuberculosis (TB) are urgently needed and may be developed by targeting essential enzymes of Mtb that sustain the pathogenicity of tuberculosis. In the present investigation, series of compounds (5a–f and 6a–f) based on a naturally occurring rugosaflavonoid moiety were evaluated by *in silico* molecular modeling studies against β-ketoacyl-ACP reductase (MabA) (PDB ID: IUZN) and pantothenate kinase (PanK) (PDB ID: 3AF3). Compounds 5a, 5c, 5d, and 6c, which had docking scores of −8.29, −8.36, −8.17 and −7.39 kcal mol^−1^, respectively, displayed interactions with MabA that were better than those of isoniazid (−6.81 kcal mol^−1^). Similarly, compounds 5a, 5c, 5d, and 6c, which had docking scores of −7.55, −7.64, −7.40 and −6.7 kcal mol^−1^, respectively, displayed interactions with PanK that were comparable to those of isoniazid (−7.64 kcal mol^−1^). Because of their docking scores, these compounds were screened *in vitro* against *Mycobacterium tuberculosis* H37Ra (Mtb) using an XRMA protocol. Among the screened compounds, the dihydrorugosaflavonoid derivatives 5a, 5c, and 5d had IC_50_ values of 12.93, 8.43 and 11.3 μg mL^−1^, respectively, and exhibited better inhibitory activity than the parent rugosaflavonoid derivatives. The rugosaflavonoid derivative 6c had an IC_50_ value of 17.57 μg mL^−1^. The synthesized compounds also displayed inhibitory activity against the Gram-positive bacteria *Bacillus subtilis* and *Staphylococcus aureus*. The present study will be helpful for the further development of these molecules into antitubercular lead candidates.

## Abstract 2

Novel drug regimens against tuberculosis (TB) are urgently needed and may be developed by targeting essential enzymes of Mtb that sustain the pathogenicity of tuberculosis. Dihydrorugosaflavonoid interacted with the active pocket of MabA and PanK.

## Graphical abstract

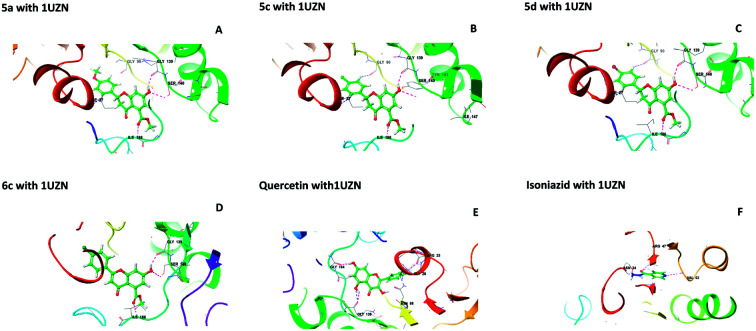

## 1. Introduction

**Scheme 1:**
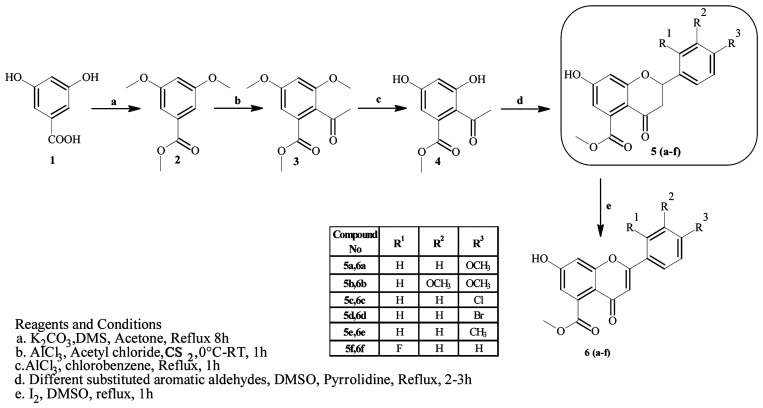
Synthesis of rugosaflavonoid and its derivatives.

*Mycobacterium tuberculosis* (Mtb) is the causative agent of tuberculosis (TB), which affected approximately 10.4 million people in 2015.^1^ The World Health Organization (WHO) introduced the DOTS (Directly Observed Treatment, Short Course) strategy, which has proven successful in effectively achieving treatment rates of higher than 90%. However, the prolonged duration (6–9 months) of the DOTS strategy and spontaneous gene mutations in pathogenic strains have led to resistance to the drugs.^2^ Hence, the emergence of cases of multi-drug resistance (MDR) and extensive drug resistance (XDR) have increased in recent years. To overcome the problems associated with this pandemic, there is an urgent need to develop effective strategies for treating and controlling TB. One strategy would comprise targeting essential enzymes of Mtb that are relevant to its survival and growth within the host cell. In this context, enzymes that participate in biosynthetic pathways represent attractive targets for the discovery of novel anti-tuberculosis agents. Among these, β-ketoacyl-ACP synthase (KAS) and pantothenate kinase (PanK) are two target enzymes that play important roles in the fatty acid synthesis (FASII) system and the biosynthetic pathway of coenzyme A (CoA), respectively.^3,4^ β-Ketoacyl-ACP reductase (MabA) comprises a complex group of enzymes responsible for the production of very-long-chain fatty acid derivatives that are the chief precursors of mycolic acids, which are the main constituents of the *M. tuberculosis* cell wall. Pantothenate kinase (PanK), on the other hand, catalyzes the ATP-dependent phosphorylation of pantothenate, which is the initial step in the universal biosynthetic pathway of coenzyme A (CoA) from pantothenic acid.^5–8^ Rugosaflavonoid is a naturally occurring flavonoid isolated by Hu *et al.*^9^ from the flower buds of the plant *Rosa rugosa*. In our ongoing research to develop simple and cost-effective synthetic methodologies for naturally occurring chromones, we have reported the first total synthesis of rugosaflavonoid and its derivatives and identified their cytotoxic potential towards breast cancer cells.^10^ Recently, Villaume *et al.*^11^ performed structure–activity relationship (SAR) studies and identified mandatory sites that are necessary for a flavone molecule to exhibit anti-TB activity. As the molecules synthesized in the present study (Scheme 1) possess the same backbone, this encouraged us to perform molecular docking studies of the active sites of β-ketoacyl-ACP reductase (MabA) (PDB ID: IUZN) and pantothenate kinase (PanK) (PDB ID: 3AF3). The results for interactions obtained by these docking studies stimulated us to carry out further *in vitro* antitubercular screening of derivatives against *M. tuberculosis* H37Ra (Mtb).

## 2. Results and discussion

### 2.1 *In silico* studies

#### 2.1.1 Molecular docking

**Fig. 1:**
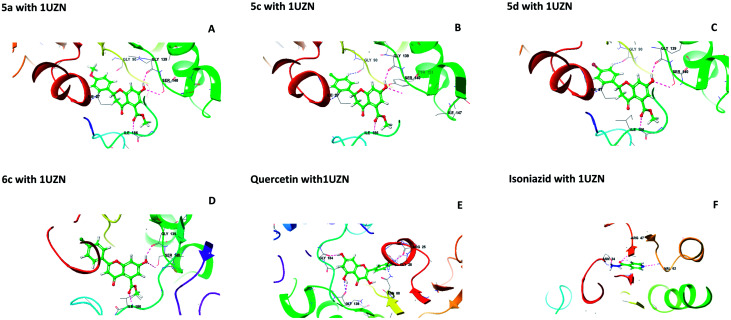
Interactions of 5a, 5c, 5d, 6c, quercetin, and isoniazid with β-ketoacyl-ACP reductase (MabA) (PDB code 1UZN).

Molecular docking studies were performed to understand the binding probability of the designed molecules. The docking studies showed that compounds 5a, 5c, 5d and 6c docked with the active pockets of 1UZN (β-ketoacyl-ACP reductase) and 3AF3 (pantothenate kinase) and interacted with the active amino acids. Compounds 5(a–f) and 6(a–f) were surrounded in the active pocket of 1UZN by Gly22, Asn24, Arg25, Gly26, Ile27, Gly28, Asn88, Ala89, Gly90, Ile138, Gly139, Ser140, Pro183, Gly184, Tyr185, Ile186, Thr188, Met190, and Thr191. Compounds 5a, 5c, 5d and 6c displayed non-bonding interactions with Gly139, Ser140, and Ile186. MabA (1UZN) contains the amino acids Ser140, Tyr153, and Lys157, which are linked to form the catalytic triad of MabA. Any mutation in the Ser140 residue results in the complete loss of enzymatic activity. Therefore, the interaction of inhibitors with Ser140 may be considered to be important for inhibition. The amino acid Gly90 has been shown to be involved in the complexation of MabA with its natural cofactor NADPH, whereas any mutation of Gly139 to Ala139 causes complete inactivation of the protein by freezing the catalytic triad into a closed form.^12^ All the active compounds were in close proximity of the active triad and established interactions with Ser140 and Gly139, as depicted in Fig. 1.

The docking scores of 5a, 5c, 5d, and 6c with 1UZN were found to be −8.296, −8.366, −8.175 and −7.398 kcal mol^−1^, respectively. The standard compound isoniazid bound in a different manner to Asn24, Arg47 and Val62 with a score of −6.813 kcal mol^−1^. Besides, we performed docking studies of quercetin, which is a ligand very well known for its biological potential. We found that quercetin interacted with Arg25, Gly28, Asn88, Gly139, and Gly184 with a docking score of −9.412 kcal mol^−1^.

**Fig. 2:**
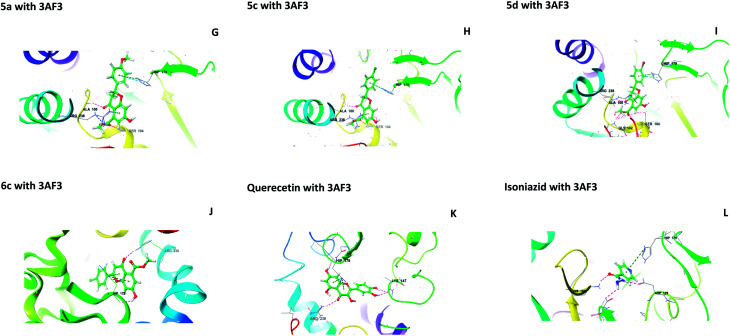
Molecular interactions of 5a, 5c, 5d, 6c, quercetin and isoniazid with pantothenate kinase (PanK) (PDB code 3AF3).

The central beta-sheet and p-loop are highly conserved in all pantothenate kinase enzymes (PanK). Differences can be seen in the surrounding loops and helix. The residues Tyr235 and Asn277 are involved in binding with pantothenate and phosphopantothenate.^13^ The active site pocket includes residues such as Hip179, Arg238, Tyr182, and Tyr177.^14^ During the molecular docking of 5(a–f) and 6(a–f) with PanK (PDB code: 3AF3), compounds 5a, 5c, 5d, and 6c displayed interactions with Ala100, Gly102, Ser104, Hip179 and Arg238 (Fig. 2).

Isoniazid interacted with Lys103, Asp129, Hip179 and Glu201. The docking scores were found to be comparable to that of isoniazid (Table 1). Quercetin interacted with Lys147, Hip179 and Arg238. Another form of PanK (PDB 4BFZ) also displayed similar kinds of interactions with these compounds in the active binding pocket (data not presented).

**Table:** Docking scores and residues of 1UZN and 3AF3 interacting with 5a–f and 6a–f

S. No.	1UZN	Docking score with 1UZN	3AF3	Docking score with 3AF3
5a	Gly139, Ser140, Ile186	−8.296	Ala100, Ser104, Hip179, Arg238	−7.553
5b	Gly139, Ser140, Ile186	−6.12	Ala100, Ser104, Hip179, Arg238	−6.83
5c	Gly139, Ser140, Ile186	−8.366	Ala100, Gly102, Ser104, Hip179, Arg238	−7.64
5d	Gly139, Ser140, Ile186	−8.175	Ala100, Gly102, Ser104, Hip179, Arg238	−7.40
5e	Ser140, Tyr153, Ile186, Thr191	−6.312	Ala100, Gly102, Ser104, Hip179, Arg238	−7.13
5f	Gly139, Ser140, Ile186	−7.913	Ala100, Gly102, Ser104, Hip179, Arg238	−7.34
6a	Asn88, Gln150, Ile186, Thr191	−6.334	Ala100, Gly102, Ser104, Arg108, Hip179	−6.052
6b	Ile27, Gly90	−6.500	Ala100, Ser104, Hip179, Arg238	−5.302
6c	Ile27, Asn88, Lys157	−7.398	Ser98, Gly102, Lys103, Hip179	−6.72
6d	Ser140, Tyr153, Thr191	−6.901	Lys103, Ser104, Hip179, Arg238	−5.533
6e	Ile27, Gly90	−6.101	Ala11, Ser104	−5.897
6f	Gly139, Ser140, Ile186	−6.788	Ala100, Gly102, Ser104, Hip179, Arg238	−6.675
Quercetin	Arg25, Gly28, Asn88, Gly139, Gly184	−9.412	Lys147, Hip179, Arg238	−7.882
Isoniazid	Asn24, Arg47, Val62	−6.813	Lys103, Asp129, Hip179, Glu201	−7.642

#### 2.1.2 Physicochemical properties and ADME predictions

An *in silico* prediction of physically significant and pharmaceutically relevant properties of the molecules was performed using QikProp. The data are presented in Table 2.

**Table:** Prediction of drug-like properties of the lead molecules by QikProp Maestro 11.2 molecular docking suite^a^

S. no.	Sample ID	*QP* log *P*_o_/*w* (−2.0 to 6.5)	*QP* log HERG (acceptable range: above −5.0)	QPP Caco (nm s^−1^) (<25 is poor, >500 is good)	*QP* log BB (−3 to 1.2)	QPP MDCK (nm s^−1^) (<25 is poor, >500 is good)	*QP* log *K*_p_ (−8.0 to −0.1)	*QP* log *K*_hsa_ (acceptable range: −1.5 to 1.5)	Percentage human oral absorption (<25% is low, >80% is high)
1	5a	2.318	−4.879	411.852	−1.037	150.21	−2.226	0.068	89.62
2	5b	2.317	−4.876	411.352	−1.138	168.31	−2.998	0.071	89.67
3	5c	2.798	−4.768	376.133	−0.787	373.182	−2.967	0.168	90.32
4	5d	2.762	−4.789	376.164	−0.767	465.314	−3.012	0.207	90.67
5	5e	2.497	−4.766	318.637	−0.982	154.32	−2.997	0.209	89.13
6	5f	2.231	−4.812	399.258	−0.813	199.87	−2.175	0.098	88.32
7	6a	2.499	−5.612	417.253	−1.040	192.330	−2.998	0.072	88.48
8	6b	2.499	−5.538	417.07	−1.141	192.141	−3.084	0.073	88.47
9	6c	2.804	−5.622	417.156	−0.798	473.982	−3.057	0.177	90.26
10	6d	2.881	−5.654	417.294	−0.791	509.985	−3.059	0.2	90.71
11	6e	2.621	−5.632	417.097	−0.984	192.252	−3.086	0.219	89.19
12	6f	2.497	−5.602	413.143	−0.870	292.220	−2.992	0.102	88.40

a Ligand CID, PubChem IDs of the lead molecules; Ligand STOCK, Updated library of natural compounds, InterBioScreen (IBS) library; Predicted IC_50_ value for blockage of HERG K^+^ channels (acceptable range: above −5.0); QPP Caco, predicted apparent Caco-2 cell permeability in nm s^−1^ (<25 is poor, >500 is good). Caco-2 cells are a model of the gut-blood barrier; *QP* log BB, predicted brain/blood partition coefficient; QPP MDCK, predicted apparent MDCK cell permeability in nm s^−1^ (<25 is poor, >500 is good). MDCK cells are considered to be a good mimic of the blood–brain barrier; *QP* log *K*_p_, predicted skin permeability; *QP* log *K*_hsa_, prediction of binding to human serum albumin (acceptable range: −1.5 to 1.5); percentage human oral absorption (<25% is low, >80% is high).

### 2.2 Biological screening

#### 2.2.1 Antitubercular activity

Because of the encouraging results of binding with β-ketoacyl-ACP reductase and pantothenate kinase, the synthesized compounds (5a–5f and 6a–6f) were subsequently screened for their *in vitro* antitubercular activity against *M. tuberculosis* H37Ra using an established XTT reduction menadione assay (XRMA). Table 3 summarises the IC_50_ and MIC values of all the compounds. Among the derivatives that were screened, compounds 5c, 5a, and 5d had IC_50_ values of 8.43, 12.93 and 11.3 μg mL^−1^, respectively, whereas compound 6c had an IC_50_ value in the range of 17.57 μg mL^−1^. All the remaining compounds exhibited IC_50_ values corresponding to a concentration of >20 μg mL^−1^.
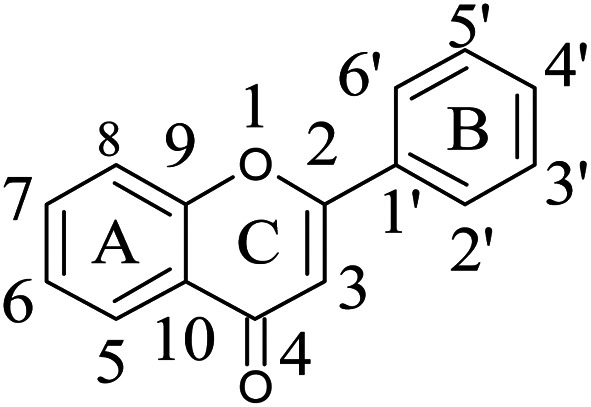


Results for *in vitro* antitubercular activity of 5a–f and 6a–f against Mtb H37Ra

**Table:** 

Sr. no.	Structure	M. W.	IC_50_ μg mL^−1^	MIC_90_ μg mL^−1^	Sr. no.	Structure	M. W.	IC_50_ μg mL^−1^	MIC_90_ μg mL^−1^
5a	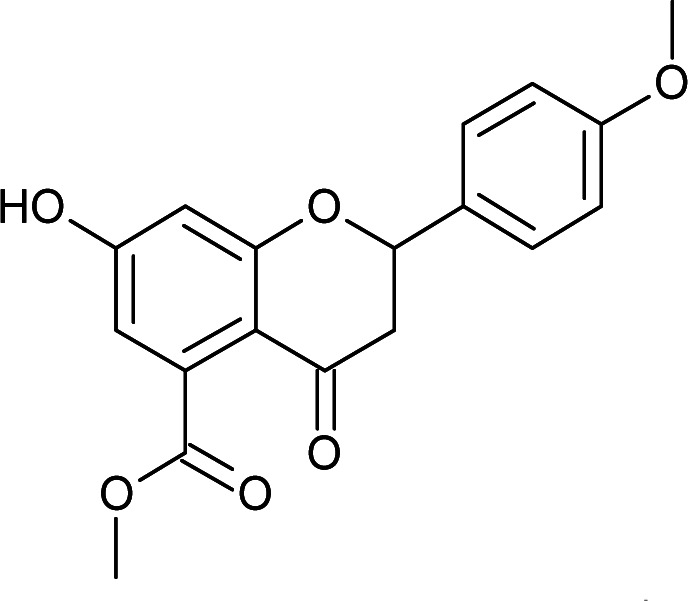	328.31	12.93	>30	6a	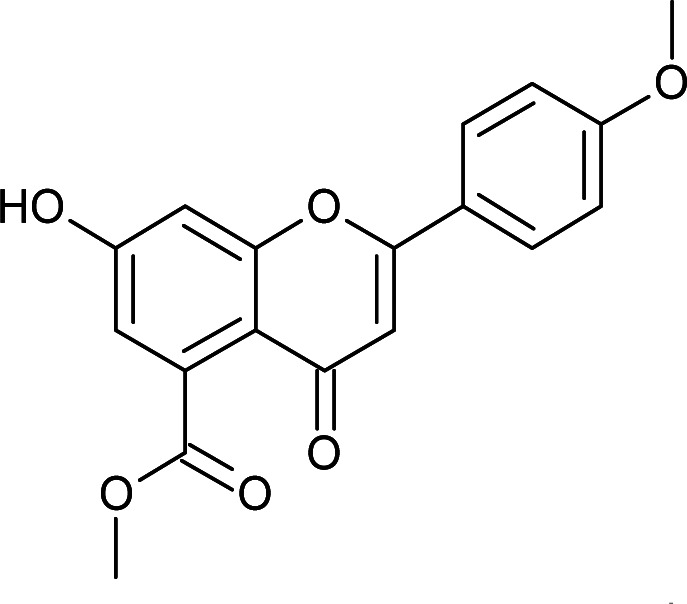	326.30	>30	>30
5b	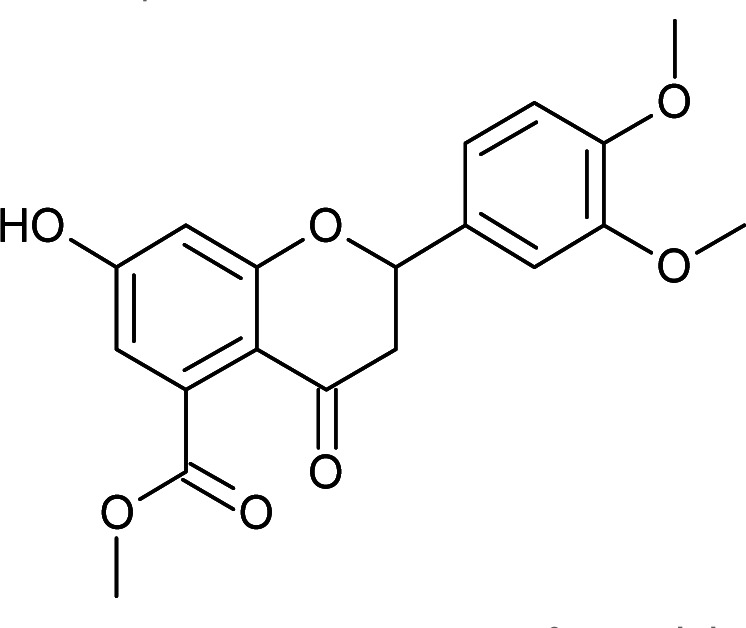	358.34	>30	>30	6b	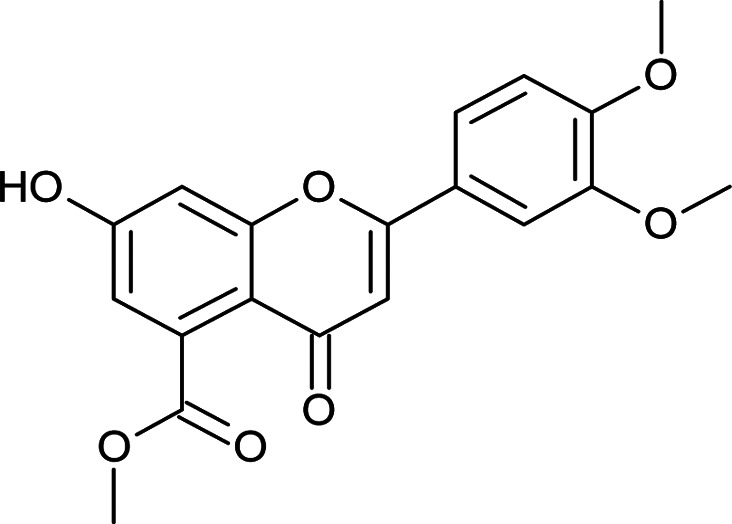	356.32	>30	>30
5c	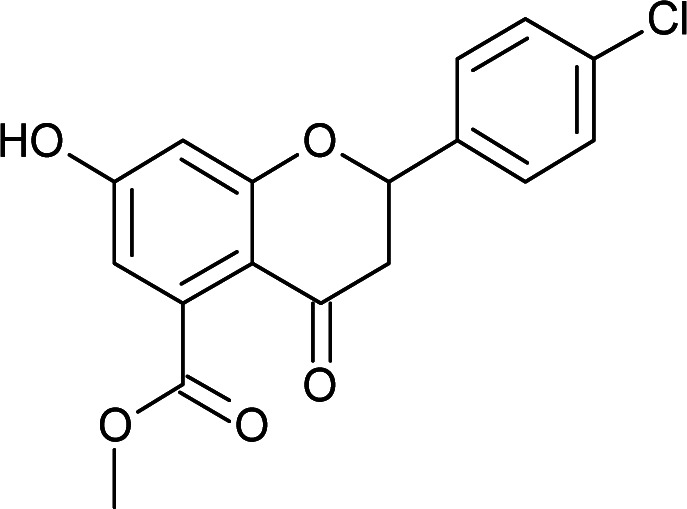	332.73	8.43	27.26	6c	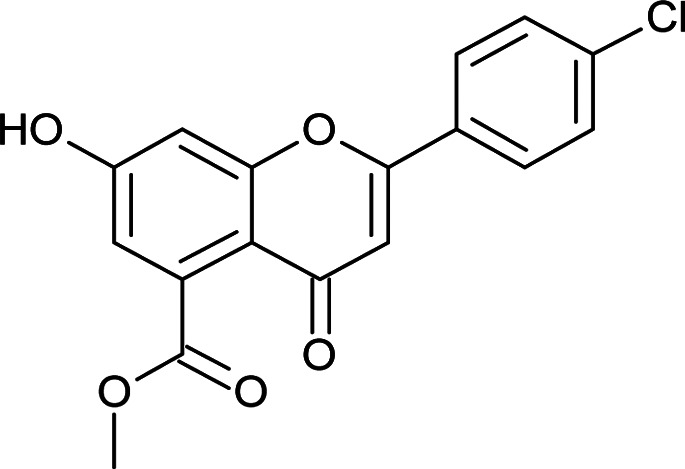	330.71	17.57	28.90
5d	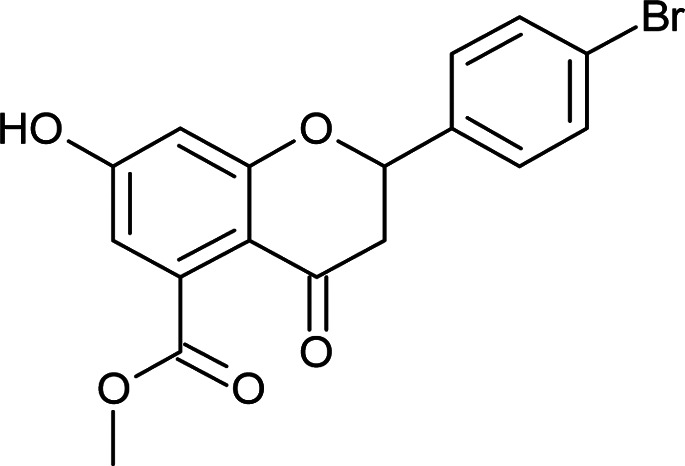	377.18	11.3	>30	6d	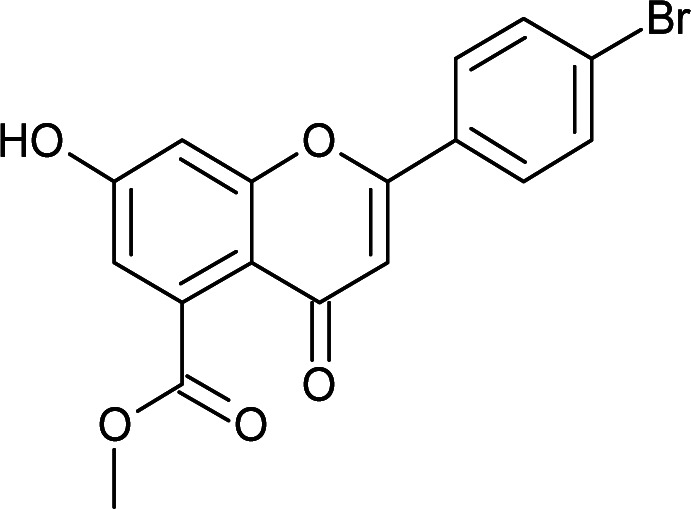	375.17	>30	>30
5e	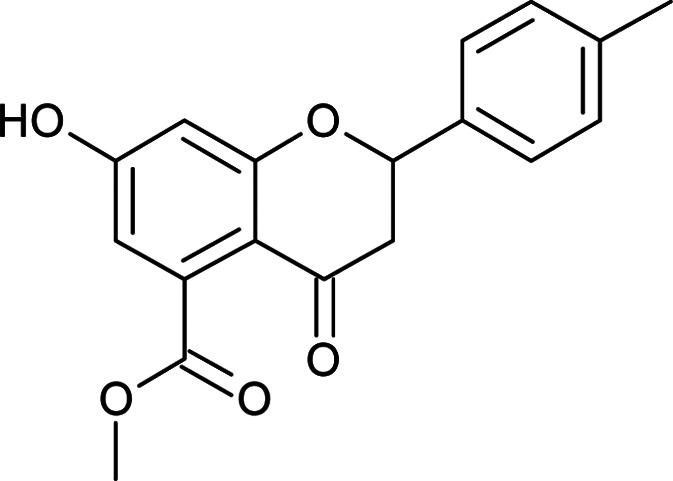	312.31	25.21	>30	6e	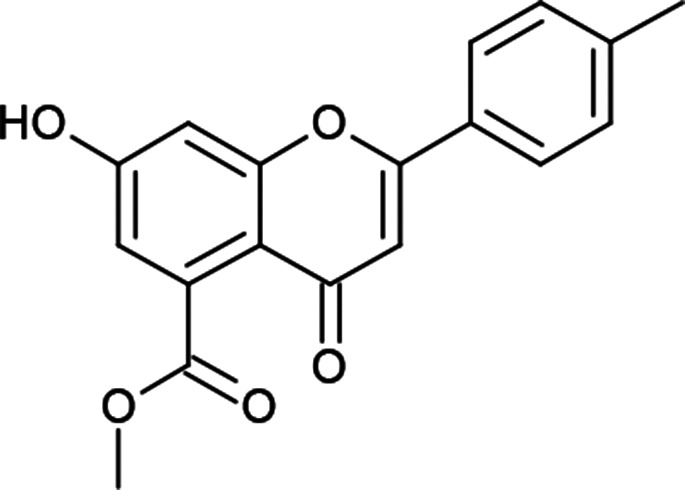	310.30	>30	>30
5f	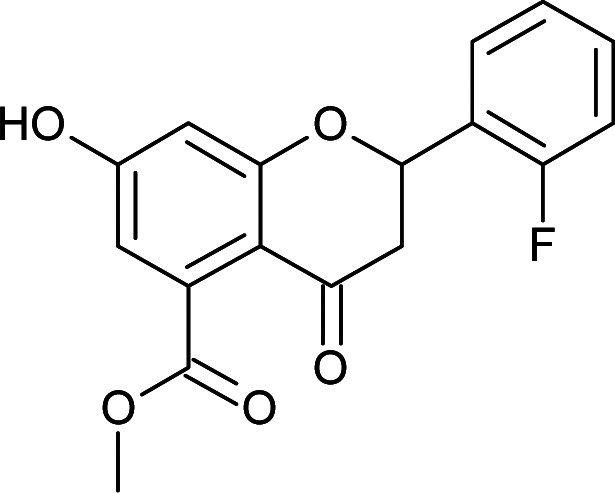	316.28	>30	>30	6f	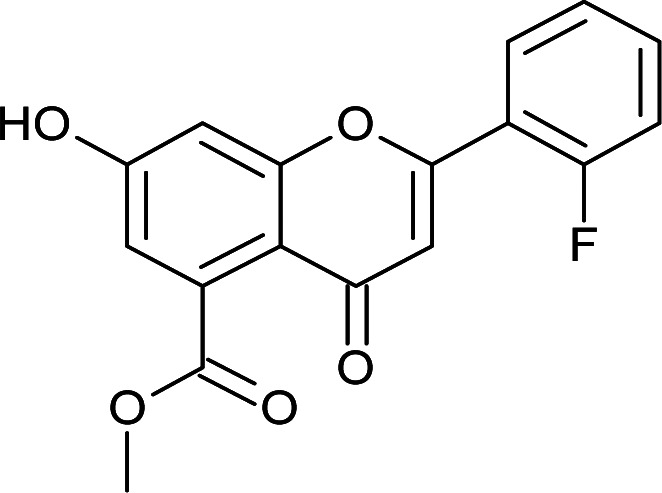	314.26	>30	>30

**Table:** 

Isoniazid	0.074	0.002
Quercetin	12.61	>30

#### 2.2.2 Basic backbone of flavone scaffold and its nomenclature

Flavone moieties contain three rings named as A, B and C. The synthesized compounds mostly have variations in ring B. The screening results for the dihydro derivatives showed variations in percentage inhibition with changes in the substituents on ring B. Amongst the tested compounds, compound 5a, which has a methoxy substituent at the 4′ position, displayed 50% inhibition at 12.93 μg mL^−1^ and 90% inhibition at >30 μg mL^−1^. The addition of another methoxy group at the 3′ position of ring B (5b) resulted in a sharp decrease in activity. The replacement of the methoxy group by a halogen atom (5c and 5d) enhanced the inhibition, and 5c and 5d exhibited 50% inhibition at 8.43 μg mL^−1^ and 11.3 μg mL^−1^, respectively. The derivative with a methyl group at the 4′ position of ring B (5e) displayed moderate activity (Table 3). Rugosaflavonoid and its derivatives (6a–f) (double bond at C2=C3) did not exhibit any inhibition except for 6c, which exhibited 90% inhibition at 28.90 μg mL^−1^. Villaume *et al.*^11^ also reported the importance of ring B when they screened naturally occurring flavones as UGM inhibitors. They observed a total loss of activity after the removal of ring B. In our case, with variations in ring B we observed declines and increases in inhibitory activity. On comparing the dihydrorugosaflavonoid derivatives with the naturally occurring molecule quercetin, we found that our compounds exhibited better inhibition than quercetin. These results indicate that dihydro derivatives of rugosaflavonoid are much better antitubercular agents than the flavone moiety of rugosaflavonoid. Besides, the results of an antibacterial assay showed that 5c and 5d displayed very good antibacterial activity against *B. subtilis*, with IC_50_ values of 6.25 μg mL^−1^ and 7.24 μg mL^−1^, respectively. When tested against *S. aureus*, 5c, 5d, 6c, 6d and 6f displayed excellent activity, with IC_50_ values of 2.77 μg mL^−1^, 5.63 μg mL^−1^, 6.45 μg mL^−1^, 8.16 μg mL^−1^ and 2.96 μg mL^−1^, respectively (Table 4). The compounds with good antibacterial activity were further tested to determine their minimum inhibitory concentration (MIC) using a dose–response curve. Compound 5c exhibited MIC values of 27.34 μg mL^−1^ and 20.44 μg mL^−1^ against *B. subtilis* and *S. aureus*, respectively. Moreover, 5d and 6c exhibited MIC values of 25.02 μg mL^−1^ and 27.53 μg mL^−1^, respectively, against *S. aureus* (Table 4). All the compounds (5a–5f and 6a–6f) exhibited IC_50_ and MIC values of >30 μg mL^−1^ against the Gram-negative strains *E. coli* and *P. aeruginosa* (Table 4). The results of the *in vitro* screening of these compounds against Gram-positive and Gram-negative bacteria demonstrated that the synthesized compounds are more active against the Gram-positive bacteria *B. subtilis* and *S. aureus* (Table 4). Finally, these results indicate that the dihydro derivatives of rugosaflavonoid are much better antitubercular agents than the flavone moiety of rugosaflavonoid and are also capable of inhibiting the growth of Gram-positive bacteria.

**Table:** *In vitro* antibacterial activity of the synthesized derivatives (5a–6f) against *E. coli*, *P. aeruginosa*, *S. aureus* and *B. subtilis*

Sample ID	Gram-positive				Gram-negative			
	*B. subtilis*		*S. aureus*		*E. coli*		*P. aeruginosa*	
	IC_50_ (μg mL^−1^)	MIC_90_ (μg mL^−1^)	IC_50_ (μg mL^−1^)	MIC_90_ (μg mL^−1^)	IC_50_ (μg mL^−1^)	MIC_90_ (μg mL^−1^)	IC_50_ (μg mL^−1^)	MIC_90_ (μg mL^−1^)
5a	>30	>30	>30	>30	>30	>30	>30	>30
5b	>30	>30	>30	>30	>30	>30	>30	>30
5c	6.25	27.34	2.77	20.44	>30	>30	>30	>30
5d	7.24	>30	5.63	25.02	>30	>30	>30	>30
5e	20.52	>30	26.44	>30	>30	>30	>30	>30
5f	19.48	>30	27.26	>30	>30	>30	>30	>30
6a	>30	>30	11.84	>30	>30	>30	>30	>30
6b	>30	>30	>30	>30	>30	>30	>30	>30
6c	17.91	>30	6.45	27.53	>30	>30	>30	>30
6d	10.27	>30	8.16	>30	>30	>30	>30	>30
6e	27.73	>30	10.07	>30	>30	>30	>30	>30
6f	>30	>30	2.96	>30	>30	>30	>30	>30

## 3. Conclusions

In the present study, we docked and synthesized dihydrorugosaflavonoid and rugosaflavonoid derivatives. The derivatives were evaluated *in silico* against Mtb β-ketoacyl-ACP reductase (MabA) (PDB ID: **1UZN**) and PanK (PDB ID: **3AF3**), and their activity was confirmed *in vitro* in *M. tuberculosis* H37Ra. The results of this integrated effort explicitly support the efficacy of the dihydrorugosaflavonoid derivatives in inhibiting MTB, which opens new avenues for the further development of these molecules into antitubercular lead candidates.

## 4. Materials and methods

### 4.1 General methods

All the chemicals used during the reactions were procured from Spectrochem, India. ^1^H NMR and ^13^C NMR spectra were recorded at room temperature using a Varian spectrometer at 400 MHz and 100 MHz, respectively. Chemical shift values are reported with reference to TMS as an internal standard. The samples were prepared by dissolving the synthesized compounds in DMSO-d_6_, and chemical shifts are expressed in *δ* (ppm) and coupling constants (*J*) in Hz. The abbreviations for the splitting patterns are as follows: s, singlet; d, doublet; t, triplet; q, quartet; m, unresolved multiplet; dd, doublet of doublets. Column chromatography was performed on Merck silica gel 60 (230–400 mesh). Analytical thin-layer chromatography was carried out on pre-coated Merck silica gel 60F_254_, and iodine was used as the developing reagent. IR spectra were recorded with a Shimadzu FTIR IR Affinity-1 spectrophotometer. CHNS analysis was performed using an Elementar Vario EL III analyzer.

### 4.2 Molecular docking

The 3D structures of MabA (PDB code: 1UZN) and PanK (PDB code: 3AF3 and 4BFZ) were chosen as ideal target proteins for docking with dihydrorugosaflavonoid and rugosaflavonoid derivatives. Naturally occurring quercetin and isoniazid were employed as standard ligands.

### 4.2.1 Protein preparation

The selected PDB files of MabA (PDB Code: 1UZN) and PanK (PDB code: 3AF3 and 4BFZ) used for docking studies were downloaded from the RCSB site (www.rcsb.org) and pre-treated prior to the docking calculations with the help of the Protein Preparation Wizard of the Maestro 11.2 program from Schrödinger. Guidelines for the use of the Protein Preparation Wizard were received from a Maestro online tutorial. These steps were followed: (i) addition of hydrogen atoms to the protein structures; (ii) assignment of bond orders; (iii) removal of crystallographic waters; (iv) regeneration of states; (v) optimization of hydrogen bonds using the PROPKA program from Schrödinger before restrained minimization using the OPLS force field; and (vi) setting the convergence of heavy atoms at the RMSD of 0.3 Å.

### 4.2.2 Ligand preparation

The dihydrorugosaflavonoid (5a–f) and rugosaflavonoid (6a–f) derivatives were selected as ligands. They were sketched using the 2D program (Ligand Preparation Wizard) of Maestro 11.2 and converted into a 3D model using the preset option. LigPrep accomplished several corrections of the ligands and provided low-energy structures with ring conformations. Energy minimization and optimization were performed using the Optimized Potential for Liquid Simulations (OPLS) force field. Subsequently, one conformation was generated for each ligand using Maestro 11.2 software from Schrödinger.

### 4.2.3 Receptor grid generation

Glide molecular docking software uses one ligand to interact with the X-ray crystal structure of the target protein for evaluation of the receptor grid for the active site. Receptor grid-dependent molecular docking helps ligands to bind in many possible conformations. Docking grids for both protein structures, namely, 1UZN and 3AF3/4BFZ were created using the receptor grid generation option of Maestro. The grid box was positioned at the center of the cognate ligands of the protein structures complexed with their natural cofactor, and the maximum length of the docked ligands was fixed at 20 Å. The scaling factor and partial charge cut-off in van der Waals radius scaling were 0.25 and 1 Å, respectively. For other parameters such as sites, constraints, rotatable groups, and excluded volumes, the default settings of Maestro 11.2 were used.

### 4.2.4 Glide molecular docking

After the generation of the ligand and protein structures and the specification of the grid at the active site of the protein, molecular docking measurements were performed. The Glide molecular docking application uses efficient computational simulation techniques for the evaluation of particular poses and ligand flexibility, such as the Glide systematic method, which is a new approach for rapid, precise molecular docking, and the resulting GScore, which is an empirical scoring function that combines several parameters. The GScore is given in kcal mol^−1^ and includes ligand–protein interaction energies, hydrophobic interactions, hydrogen bonds, internal energies, pi–pi stacking interactions, root mean square deviation (RMSD) and desolvation. The Glide module of the XP visualizer was used to analyze the specific ligand–protein interactions. The dihydrorugosaflavonoids, rugosaflavonoids, quercetin and the standard isoniazid were docked with the 3D structures of MabA and PanK with the help of Glide. The best-fit compounds for each target were identified by their optimal thermodynamic energy values, types of interactions, bonding potential, and conformations.^15,16^

### 4.2.5 Studies of ADME properties

The QikProp tool from Schrödinger (2017) was used to calculate, and provided information about, the absorption, distribution, metabolism, excretion, and toxicity (ADME/T) properties of the ligand molecules. It provided information such as *QP* log *P*_o_/*w*, *QP* log BB, overall CNS activity, Caco-2 and MDCK cell permeability, log *K*_hsa_ for binding to human serum albumin, percentage human oral absorption, *etc.*^17^

### 4.3 Chemistry

#### 4.3.1 Synthesis of methyl 3,5-dimethoxybenzoate (2)

This was prepared as per the method reported by McNulty and Mcleod.^18^ In brief, 3,5-dihydroxybenzoic acid (6 mmol) in dry acetone was taken. To this mixture, K_2_CO_3_ (2.5 mmol) was added under stirring at 40 °C for 15 min, and stirring was continued at 60 °C for 10 min. Then, dimethyl sulfate (2.2 mmol) was added dropwise over a period of 30 min, and the temperature was increased slowly to 80 °C. The reaction mixture was allowed to reflux for 6 h. The progress of the reaction was monitored by TLC. After completion, the reaction mixture was allowed to cool to room temperature and filtered through a celite bed. The filtered mixture was concentrated to obtain the crude product, which was slowly poured onto crushed ice with constant stirring to obtain a solid. The solid that was obtained was filtered and dried to give methyl 3,5-dimethoxybenzoate (2) in 91% yield.

#### 4.3.2 Synthesis of methyl 2-acetyl-3,5-dimethoxybenzoate (3)

Methyl 3,5-dimethoxybenzoate (2) (5 mmol) was mixed with acetyl chloride (25 mmol) and carbon disulfide (2 mL) under dry N_2_ in an ice bath.^19^ To the reaction mixture AlCl_3_ (15 mmol) was added under vigorous stirring. The reaction was allowed to stir for 15 min. The progress of the reaction was monitored by TLC. After completion, the reaction mixture was quenched with ice and extracted with ethyl acetate. The organic layer was separated, dried over sodium sulfate and concentrated to give the crude product, which was purified by column chromatography (hexane–ethyl acetate, 70 : 30) to obtain methyl 2-acetyl-3,5-dimethoxybenzoate (3) in 52% yield.

#### 4.3.3 Synthesis of methyl 2-acetyl-3,5-dihydroxybenzoate (4)

Methyl 2-acetyl-3,5-dimethoxybenzoate (3) (4 mmol) was added to chlorobenzene, and AlCl_3_ (10 mmol) was added slowly at room temperature.^20^ The reaction mixture was heated to reflux for 1 h. The progress of the reaction was monitored by TLC. After completion, the reaction mixture was cooled to room temperature and hydrolysed using 1 N HCl. The reaction mixture was extracted with ethyl acetate. The organic layer was separated, dried over sodium sulfate and concentrated to obtain the crude product, which was purified by column chromatography (hexane–ethyl acetate, 80 : 20) to give clean methyl 2-acetyl-3,5-dihydroxybenzoate (4) in 68% yield.

#### 4.3.4 General procedure for the synthesis of methyl 7-hydroxy-2-(substituted phenyl)-4-oxo-3,4-dihydro-2*H*-chromene-5-carboxylates (5a–f)

Methyl 2-acetyl-3,5-dihydroxybenzoate (4) (4.7 mmol) in DMSO was mixed with different substituted aromatic aldehydes (4.7 mmol), I_2_ (0.23 mmol) and pyrrolidine (2.3 mmol) as per the reported procedure,^21^ and the reaction mixture was allowed to reflux for 8 h. The progress of the reaction was monitored by TLC. After completion, the mixture was cooled to room temperature and quenched with water. The aqueous layer was extracted with ethyl acetate. The organic layer was separated and washed with a brine solution. The organic layer was dried and concentrated to give the crude product, which was purified by column chromatography using hexane:ethyl acetate as the solvent system to obtain the products 5a–f.

#### 4.3.5 Methyl 7-hydroxy-2-(4-methoxyphenyl)-4-oxo-3,4-dihydro-2*H*-chromene-5-carboxylate (5a)

Yellow solid (48%), *R*_f_ 0.46 (EtOAc/hexane, 3 : 7); mp: 201 °C; IR (KBr, cm^−1^): 3412 (OH), 1707 (C=O); ^1^H NMR (400 MHz, DMSO-d_6_): *δ* 10.94 (s, 1H, OH), 7.44 (d, *J* = 8.4 Hz, 2H, 2′-H, 6′-H), 6.96 (d, *J* = 8.8 Hz, 2H, 3′-H, 5′-H), 6.42 (s, 2H, 6-H, 8-H), 5.55 (dd, *J* = 1.6, 12 Hz, 1H, 2-H), 3.75 (s, 3H, OCH_3_), 3.69 (s, 3H, OCH_3_), 3.16 (q, *J* = 3.2, 13.2 Hz, 1H, 3-H), 2.67 (dd, *J* = 2.4, 14.8 Hz, 1H, 3-H); ^13^C NMR (100 MHz, DMSO-d_6_): *δ* 189.49, 169.50, 164.36, 163.66, 159.92, 136.42, 131.76, 131.11, 128.73, 114.32, 110.82, 109.76, 107.64, 107.55, 79.19, 52.78, 52.46, 43.56; LCMS (ESI): *m*/*z* calculated for C_18_H_16_O_6_: 328.31, found: 329.10. Elemental analysis: calculated for C_18_H_16_O_6_: C, 65.84, H, 4.90; found: C, 65.91, H, 4.96.

#### 4.3.6 Methyl 2-(3,4-dimethoxyphenyl)-7-hydroxy-4-oxo-3,4-dihydro-2*H*-chromene-5-carboxylate (5b)

Yellow solid (41%), *R*_f_ 0.5 (EtOAc/hexane, 3 : 7); mp: 218 °C; IR (KBr, cm^−1^): 3477 (OH), 1732 (C=O); ^1^H NMR (400 MHz, DMSO-d_6_): *δ* 10.97 (s, 1H, OH), 7.13 (s, 1H, 2′-H), 7.03 (d, *J* = 8.0 Hz, 1H, 6′-H), 6.96 (d, *J* = 8.0 Hz, 1H, 5′-H), 6.43 (s, 2H, 6-H, 8-H), 5.52 (dd, *J* = 2.8, 12.8 Hz, 1H, 2-H), 3.75 (s, 9H, OCH_3_), 3.21 (q, *J* = 4, 13.2 Hz, 1H, 3-H), 2.67 (dd, *J* = 2.8, 14.4 Hz, 1H, 3-H); ^13^C NMR (100 MHz, DMSO-d_6_): *δ* 189.53, 169.50, 164.36, 163.64, 149.51, 149.18, 136.40, 131.46, 119.79, 111.98, 111.09, 110.84, 109.78, 104.21, 79.51, 56.03, 56.03, 52.78, 43.66; LCMS (ESI): *m*/*z* calculated for C_19_H_18_O_7_: 358.34, found: 359.10. Elemental analysis: calculated for C_19_H_18_O_7_: C, 63.67, H, 5.05; found: C, 63.62, H, 4.99.

#### 4.3.7 Methyl 2-(4-chlorophenyl)-7-hydroxy-4-oxo-3,4-dihydro-2*H*-chromene-5-carboxylate (5c)

Yellow solid (40%), *R*_f_ 0.68 (EtOAc/hexane, 3 : 7); mp: 198 °C; IR (KBr, cm^−1^): 3361 (OH), 1735 (C=O); ^1^H NMR (400 MHz, DMSO-d_6_): *δ* 10.96 (s, 1H, OH), 7.53 (d, *J* = 8.8 Hz, 2H, 3′-H, 5′-H), 7.46 (d, *J* = 8.4 Hz, 2H, 2′-H, 6′-H), 6.44 (s, 1H, 8-H), 6.41 (s, 1H, 6-H), 5.63 (dd, *J* = 3.2, 12.8 Hz, 1H, 2-H), 3.74 (s, 3H, OCH_3_), 3.09 (q, *J* = 4, 12.8 Hz, 1H, 3-H), 2.72 (dd, *J* = 3.2, 13.6 Hz, 1H, 3-H); ^13^C NMR (100 MHz, DMSO-d_6_): *δ* 189.09, 169.48, 164.51, 163.48, 138.28, 136.50, 133.64, 131.72, 129.91, 129.09, 129.09, 110.87, 110.02, 104.28, 78.69, 52.86, 43.63; LCMS (ESI): *m*/*z* calculated for C_17_H_13_ClO_5_: 332.73, found: 333.0. Elemental analysis: calculated for C_17_H_13_ClO_5_: C, 61.36, H, 3.93; found: C, 61.30, H, 3.87.

#### 4.3.8 Methyl 2-(4-bromophenyl)-7-hydroxy-4-oxo-3,4-dihydro-2*H*-chromene-5-carboxylate (5d)

Yellow solid (40%), *R*_f_ 0.67 (EtOAc/hexane, 3 : 7); mp: 208 °C; IR (KBr, cm^−1^): 3361 (OH), 1735 (C=O); ^1^H NMR (400 MHz, DMSO-d_6_): *δ* 10.97 (s, 1H, OH), 7.60 (d, *J* = 8.8 Hz, 2H, 3′-H, 5′-H), 7.46 (d, *J* = 8.4 Hz, 2H, 2′-H, 6′-H), 6.44 (s, 1H, 8-H), 6.41 (s, 1H, 6-H), 5.62 (dd, *J* = 2.4, 12.8 Hz, 1H, 2-H), 3.74 (s, 3H, OCH_3_), 3.08 (q, *J* = 4.4, 12.8 Hz, 1H, 3-H), 2.72 (dd, *J* = 2.8, 14.4 Hz, 1H, 3-H); ^13^C NMR (100 MHz, DMSO-d_6_): *δ* 189.05, 169.48, 164.53, 163.46, 138.69, 136.50, 132.01, 132.01, 129.34, 129.34, 122.23, 110.87, 110.03, 104.28, 78.72, 52.86, 43.59; LCMS (ESI): *m*/*z* calculated for C_17_H_13_BrO_5_: 377.18, found: 377.8. Elemental analysis: calculated for C_17_H_13_BrO_5_: C, 54.13, H, 3.47; found: C, 54.08, H, 3.43.

#### 4.3.9 Methyl 7-hydroxy-2-(4-methylphenyl)-4-oxo-3,4-dihydro-2*H*-chromene-5-carboxylate (5e)

Yellow solid (40%), *R*_f_ 0.7 (EtOAc/hexane, 3.5 : 6.5); mp: 198 °C; IR (KBr, cm^−1^): 3367 (OH), 1739 (C=O); ^1^H NMR (400 MHz, DMSO-d_6_): *δ* 10.97 (s, 1H, OH), 7.40 (d, *J* = 8 Hz, 2H, 2′-H, 6′-H), 7.23 (d, *J* = 7.6 Hz, 2H, 3′-H, 5′-H), 6.45 (s, 1H, 8-H), 6.43 (s, 1H, 6-H), 5.58 (dd, *J* = 2.4, 12.4 Hz, 1H, 2-H), 3.77 (s, 3H, OCH_3_), 3.13 (q, *J* = 4.4, 12.8 Hz, 1H, 3-H), 2.71 (dd, *J* = 2.4, 14.8 Hz, 1H, 3-H), 2.32 (s, 3H, CH_3_); ^13^C NMR (100 MHz, DMSO-d_6_): *δ* 189.34, 169.48, 164.38, 163.59, 138.39, 136.42, 136.21, 129.51, 129.51, 127.10, 127.10, 110.84, 109.80, 104.19, 79.30, 52.78, 43.62, 21.24; LCMS (ESI): *m*/*z* calculated for C_18_H_16_O_5_: 312.31, found: 313.05. Elemental analysis: calculated for C_18_H_16_O_5_: C, 69.21, H, 5.15; found: C, 69.27, H, 5.21.

#### 4.3.10 Methyl 2-(2-fluorophenyl)-7-hydroxy-4-oxo-3,4-dihydro-2*H*-chromene-5-carboxylate (5f)

Yellow solid (40%), *R*_f_ 0.68 (EtOAc/hexane, 3 : 7); mp: 195 °C; IR (KBr, cm^−1^): 3461 (OH), 1735 (C=O); ^1^H NMR (400 MHz, DMSO-d_6_): *δ* 11.03 (s, 1H, OH), 7.64–7.68 (m, 1H, 3′-H), 7.45–7.48 (m, 1H, 4′-H), 7.26–7.32 (m, 2H, 5′-H, 6′-H), 6.46 (s, 2H, 6-H, 8-H), 5.85 (dd, *J* = 2.4, 13.2 Hz, 1H, 2-H), 3.78 (s, 3H, OCH_3_), 3.23 (q, *J* = 3.2, 14 Hz, 1H, 3-H), 2.73 (dd, *J* = 2.4, 14 Hz, 1H, 3-H); ^13^C NMR (100 MHz, DMSO-d_6_): *δ* 188.85, 169.40, 164.46, 163.43, 161.32, 158.86, 136.50, 131.40, 128.97, 125.90, 125.24, 116.32, 110.70, 104.15, 73.95, 52.82, 42.27; LCMS (ESI): *m*/*z* calculated for C_17_H_13_FO_5_: 316.28, found: 317.05. Elemental analysis: calculated for C_17_H_13_FO_5_: C, 64.55, H, 4.13; found: C, 64.49, H, 4.20.

#### 4.3.11 General procedure for the synthesis of methyl 7-hydroxy-2-(substituted phenyl)-4-oxo-4*H*-chromene-5-carboxylates (6a–f)

I_2_ (0.15 mmol) was added to methyl 7-hydroxy-2-(substituted phenyl)-4-oxo-3,4-dihydro-2*H*-chromene-5-carboxylates (5a–f) (3 mmol) in DMSO (10 mL), and the mixture was refluxed for 1 h. The progress of the reaction was observed by TLC. After completion, the reaction mixture was cooled to room temperature and quenched with water. The aqueous layer was extracted with ethyl acetate. The organic layer was separated, dried over sodium sulfate and concentrated, and the crude product was obtained, which was purified by column chromatography (hexane–ethyl acetate, 40 : 60) to obtain methyl 7-hydroxy-2-(substituted phenyl)-4-oxo-4*H*-chromene-5-carboxylates 6(a–f) in 60% yield.

#### 4.3.12 Methyl 7-hydroxy-2-(4-methoxyphenyl)-4-oxo-4*H*-chromene-5-carboxylate (6a)

Mp: 226–228 °C; IR (KBr, cm^−1^): 3446 (OH), 1735 (C=O), 1624 (C=O); ^1^H NMR (400 MHz, DMSO-d_6_): *δ* 11.14 (s, 1H, OH), 8.04 (d, *J* = 7.2 Hz, 2H, 2′-H, 6′-H), 7.12 (d, *J* = 7.2 Hz, 2H, 3′-H, 5′-H), 7.10 (d, *J* = 2 Hz, 1H, 6-H), 6.82 (d, *J* = 1.6 Hz, 1H, 8-H), 6.79 (s, 1H, 3-H), 3.86 (s, 3H, OCH_3_), 3.81 (s, 3H, OCH_3_); ^13^C NMR (100 MHz, DMSO-d_6_): *δ* 175.68, 169.19, 162.54, 162.39, 158.55, 157.70, 134.55, 128.59, 127.11, 123.49, 114.98, 114.81, 113.94, 113.55, 105.58, 104.25, 55.97, 52.86; LCMS (ESI): *m*/*z* calculated for C_18_H_14_O_6_: 326.3, found: 327.0. Elemental analysis: calculated for C_18_H_14_O_6_: C, 66.25, H, 4.32; found: C, 66.31, H, 4.28.

#### 4.3.13 Methyl 2-(3,4-dimethoxyphenyl)-7-hydroxy-4-oxo-4*H*-chromene-5-carboxylate (6b)

Mp: 233–236 °C; IR (KBr, cm^−1^): 3444 (OH), 1737 (C=O), 1627 (C=O); ^1^H NMR (400 MHz, DMSO-d_6_): *δ* 11.08 (s, 1H, OH), 7.63 (d, *J* = 8 Hz, 1H, 6′-H), 7.52 (s, 1H, 2′-H), 7.13 (m, 2H, 5′-H, 6-H), 6.84 (s, 1H, 8-H), 6.79 (s, 1H, 3-H), 3.85 (s, 3H, OCH_3_), 3.81 (s, 3H, OCH_3_), 3.78 (s, 3H, OCH_3_); ^13^C NMR (100 MHz, DMSO-d_6_): *δ* 175.74, 169.19, 162.48, 162.36, 157.74, 152.34, 149.47, 134.51, 123.59, 120.29, 113.53, 113.39, 112.14, 109.83, 105.89, 104.34, 56.32, 56.17, 52.87; LCMS (ESI): *m*/*z* calculated for C_19_H_16_O_7_: 356.32, found: 357.0. Elemental analysis: calculated for C_19_H_16_O_7_: C, 64.04, H, 4.52; found: C, 64.16, H, 4.59.

#### 4.3.14 Methyl 2-(4-chlorophenyl)-7-hydroxy-4-oxo-4*H*-chromene-5-carboxylate (6c)

Mp: 262–268 °C; IR (KBr, cm^−1^): 3645 (OH), 1714 (C=O), 1697 (C=O); ^1^H NMR (400 MHz, DMSO-d_6_): *δ* 11.19 (s, 1H, OH), 8.07 (d, *J* = 8.8 Hz, 2H, 3′-H, 5′-H), 7.60 (d, *J* = 8.4 Hz, 2H, 2′-H, 6′-H), 7.08 (s, 1H, 6-H), 6.89 (s, 1H, 8-H), 6.80 (s, 1H, 3-H), 3.78 (s, 3H, OCH_3_); ^13^C NMR (100 MHz, DMSO-d_6_): *δ* 175.75, 169.06, 162.63, 161.24, 157.79, 136.99, 134.60, 130.28, 129.62, 129.62, 128.60, 128.60, 113.86, 113.36, 107.48, 104.32, 52.91; LCMS (ESI): *m*/*z* calculated for C_17_H_11_ClO_5_: 330.71, found 331.0. Elemental analysis: calculated for C_17_H_11_ClO_5_: C, 61.73, H, 3.34; found: C, 61.67, H, 3.28.

#### 4.3.15 Methyl 2-(4-bromophenyl)-7-hydroxy-4-oxo-4*H*-chromene-5-carboxylate (6d)

Mp: 275–277 °C; IR (KBr, cm^−1^): 3564 (OH), 1737 (C=O), 1627 (C=O); ^1^H NMR (400 MHz, DMSO-d_6_): *δ* 11.18 (s, 1H, OH), 8.0 (d, *J* = 8 Hz, 2H, 3′-H, 5′-H), 7.75 (d, *J* = 8 Hz, 2H, 2′-H, 6′-H), 7.07 (s, 1H, 3-H), 6.90 (s, 1H, 6-H), 6.81 (s, 1H, 8-H), 3.78 (s, 3H, OCH_3_); ^13^C NMR (100 MHz, DMSO-d_6_): *δ* 175.90, 169.90, 162.70, 161.52, 157.84, 134.62, 132.62, 132.62, 130.59, 128.76, 128.76, 126.01, 113.93, 113.35, 107.41, 104.38, 53.03; LCMS (ESI): *m*/*z* calculated for C_17_H_11_BrO_5_: 375.17, found: 376.9, 378.9. Elemental analysis: calculated for C_17_H_11_BrO_5_: C, 54.42, H, 2.95; found: C, 54.48, H, 2.95.

#### 4.3.16 Methyl 7-hydroxy-2-(4-methylphenyl)-4-oxo-4*H*-chromene-5-carboxylate (6e)

Mp: 240–242 °C; IR (KBr, cm^−1^): 3516 (OH), 1737 (C=O), 1627 (C=O); ^1^H NMR (400 MHz, DMSO-d_6_): *δ* 11.18 (s, 1H, OH), 7.95 (d, *J* = 8.0 Hz, 2H, 2′-H, 6′-H), 7.38 (d, *J* = 8.0 Hz, 2H, 3′-H, 5′-H), 7.10 (s, 1H, 6-H), 6.84 (s, 1H, 8-H), 6.83 (s, 1H, 3-H), 3.81 (s, 3H, OCH_3_), 2.39 (s, 3H, CH_3_); ^13^C NMR (100 MHz, DMSO-d_6_): *δ* 175.76, 169.14, 162.49, 157.78, 142.42, 134.58, 130.25, 130.14, 130.14, 128.55, 126.68, 126.68, 113.68, 113.43, 106.46, 104.27, 52.88, 21.51; LCMS (ESI): *m*/*z* calculated for C_18_H_14_O_5_: 310.3, found: 311.0. Elemental analysis: calculated for C_18_H_14_O_5_: C, 69.66, H, 4.54; found: C, 69.61, H, 4.49.

#### 4.3.17 Methyl 2-(2-fluorophenyl)-7-hydroxy-4-oxo-4*H*-chromene-5-carboxylate (6f)

Mp: 222–225 °C; IR (KBr, cm^−1^): 3645 (OH), 1732 (C=O), 1697 (C=O); ^1^H NMR (400 MHz, DMSO-d_6_): *δ* 11.22 (s, 1H, OH), 8.02–8.06 (m, 1H, 3′-H), 7.63–7.70 (m, 1H, 4′-H), 7.41–7.5 (m, 2H, 5′-H, 6′-H), 7.06 (s, 1H, 6-H), 6.86 (s, 1H, 8-H), 6.65 (s, 1H, 3-H), 3.81 (s, 3H, OCH_3_); ^13^C NMR (100 MHz, DMSO-d_6_): *δ* 185.83, 169.39, 164.45, 163.43, 161.07, 159.11, 136.50, 131.36, 128.96, 128.93, 125.21, 116.30, 116.12, 110.71, 110.07, 104.15, 52.81; LCMS (ESI): *m*/*z* calculated for C_17_H_11_FO_5_: 314.26, found: 315.0. Elemental analysis: calculated for C_17_H_11_FO_5_: C, 64.96, H, 3.52; found: C, 64.91, H, 3.56.

## 5. Biological activity

All the chemicals used for the activity study were procured from Sigma-Aldrich, USA.

### 5.1 Evaluation of *in vitro* antimycobacterial activity

#### 5.1.1 Cultivation of mycobacteria

The *Mycobacterium tuberculosis* H37Ra (ATCC 25177) strain was obtained from AstraZeneca, India. The stock culture was maintained at −80 °C and subcultured once in liquid *M. phlei* medium, which contained 0.5 g KH_2_PO_4_, 0.25 g trisodium citrate, 60 mg MgSO_4_, 0.5 g asparagine and 2 mL glycerol in distilled water (100 mL), followed by adjustment of the pH to 6.6. Stock cultures of bacilli were first grown in *M. phlei* medium at 37 °C at 150 rpm for at least 8–10 days before an OD of 1 at 620 nm was recorded.

#### 5.1.2 Antitubercular activity

Stock solutions (10 mg mL^−1^) of all the newly synthesized compounds were prepared in DMSO and were evaluated for their *in vitro* antitubercular activity against *M. tuberculosis* H37Ra (ATCC 25177) in a liquid medium using an established XTT reduction menadione assay (XRMA) method.^22^ In brief, 2.5 μL of a test solution (30–0 μg mL^−1^) was added to 248.5 μL of *M. phlei* medium containing bacilli and incubated at 37 °C for 8 days. Post-incubation, the XRMA was carried out to estimate the number of viable cells present in different wells of the assay plate. The absorbance was recorded with a microplate reader (SpectraMax Plus 384, Molecular Devices, Inc.) using a 470 nm filter against a blank prepared from cell-free wells. The absorbance displayed by cells treated with the vehicle alone was taken as representing 100% cell growth. The MIC and IC_50_ values of the compounds were calculated using Origin 6 software. The percentage inhibition was calculated using the following equation: percentage inhibition = [(absorbance of control − absorbance of test sample)/(absorbance of control − absorbance of blank)] × 100, where control denotes the medium with bacilli together with the vehicle and blank denotes the cell-free medium. A drug in clinical use, namely, isoniazid, was used as a reference.

#### 5.1.3 Antibacterial activity

To investigate the specificity of the synthesised derivatives 5a–f and 6a–f, all the compounds were screened for their antibacterial activity in 96-well plates against four bacterial strains (Gram-negative strains: *Escherichia coli* (NCIM 2065; ATCC 8739) and *Pseudomonas aeruginosa* (NCIM 5029; ATCC 27853); Gram-positive strains: *Staphylococcus aureus* (NCIM 2901; ATCC 29737) and *Bacillus subtilis* (NCIM 2920; ATCC 6051)). All four strains were grown in Luria–Bertani medium from HiMedia, India. Once the culture reached an OD_620_ of 1, it was used to test antibacterial activity. Bacterial cultures with an OD_620_ of 0.1 were treated with the synthesized compounds at different concentrations (30, 10, and 3 μg mL^−1^) and incubated for 8 h at 37 °C. The post-incubation OD_620_ was measured for both Gram-positive and Gram-negative bacteria.

## Conflicts of interest

There is no conflict of interest among the authors.

## Supplementary Material

RA-008-C8RA00636A-s001
