# Supplementary material for: Molecular modeling studies and in vitro screening of dihydrorugosaflavonoid and its derivatives against Mycobacterium tuberculosis
Source: RSC Adv. 2018 Mar 16;8(19):10634–43. doi: 10.1039/c8ra00636a (PMC9078922; doi:10.1039/c8ra00636a)
Supplement: RA-008-C8RA00636A-s001 [file RA-008-C8RA00636A-s001.pdf]

Electronic Supplementary Material (ESI) for RSC Advances.
This journal is © The Royal Society of Chemistry 2018

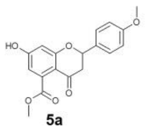

5a

Mol. Formula: $C_{18}H_{16}O_6$

Mol. Weight: 328.31

LCMS REPORT

Column : X-Select CSH C18 (3.0*50) mm 2.5u
Mobile Phase: A: 0.05% Formic acid in water + 5%ACN
B: 0.05% Formic acid in Acetonitrile
Inj Volume: 2.0µL
Flow Rate: 1.2 mL/minute
Gradient program:
0% B to 98 % B in 2.0 minute, hold till 3.0 min, at 3.2 min B conc is 0 % up to 4.0 min

Chromatogram

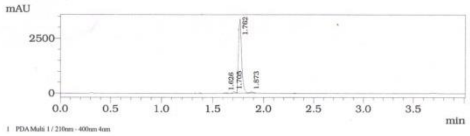

MS Chromatogram

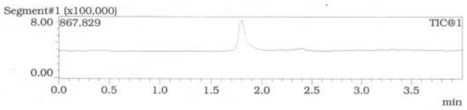

Ret. Time : 1.717 min Base Peak:326.70(1250) Polarity:Pos Segment1 - Event1

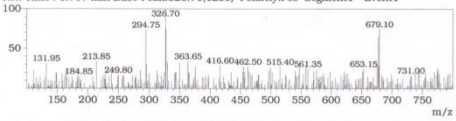

Ret. Time : 1.800 min Base Peak:329.10(224531) Polarity:Pos Segment1 - Event1

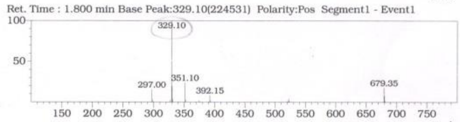

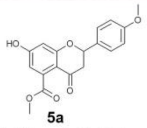

5a

Mol. Formula: $C_{18}H_{16}O_6$

Mol. Weight: 328.31

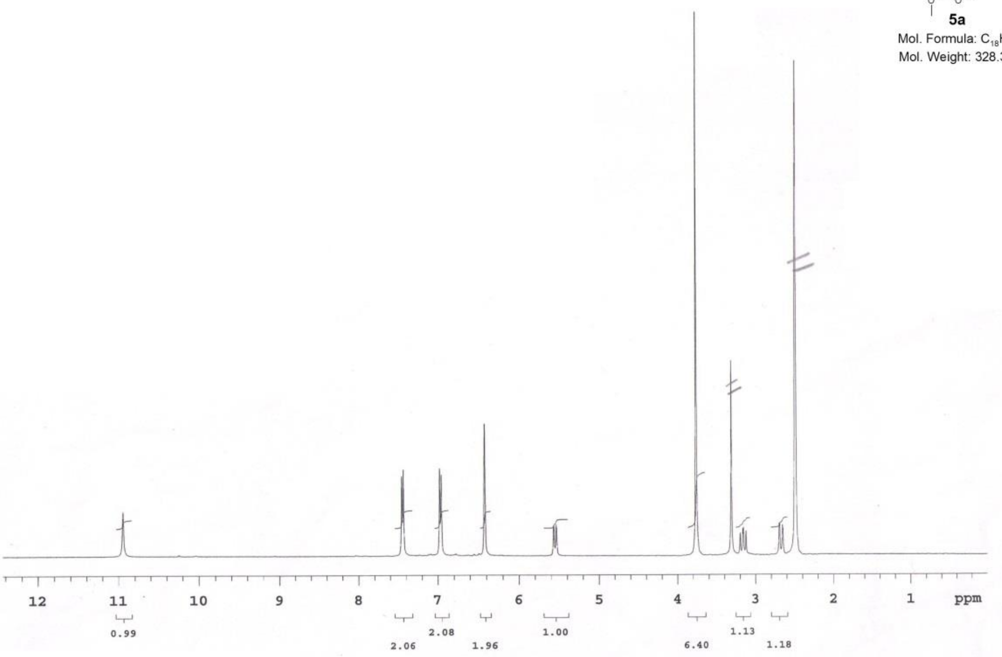

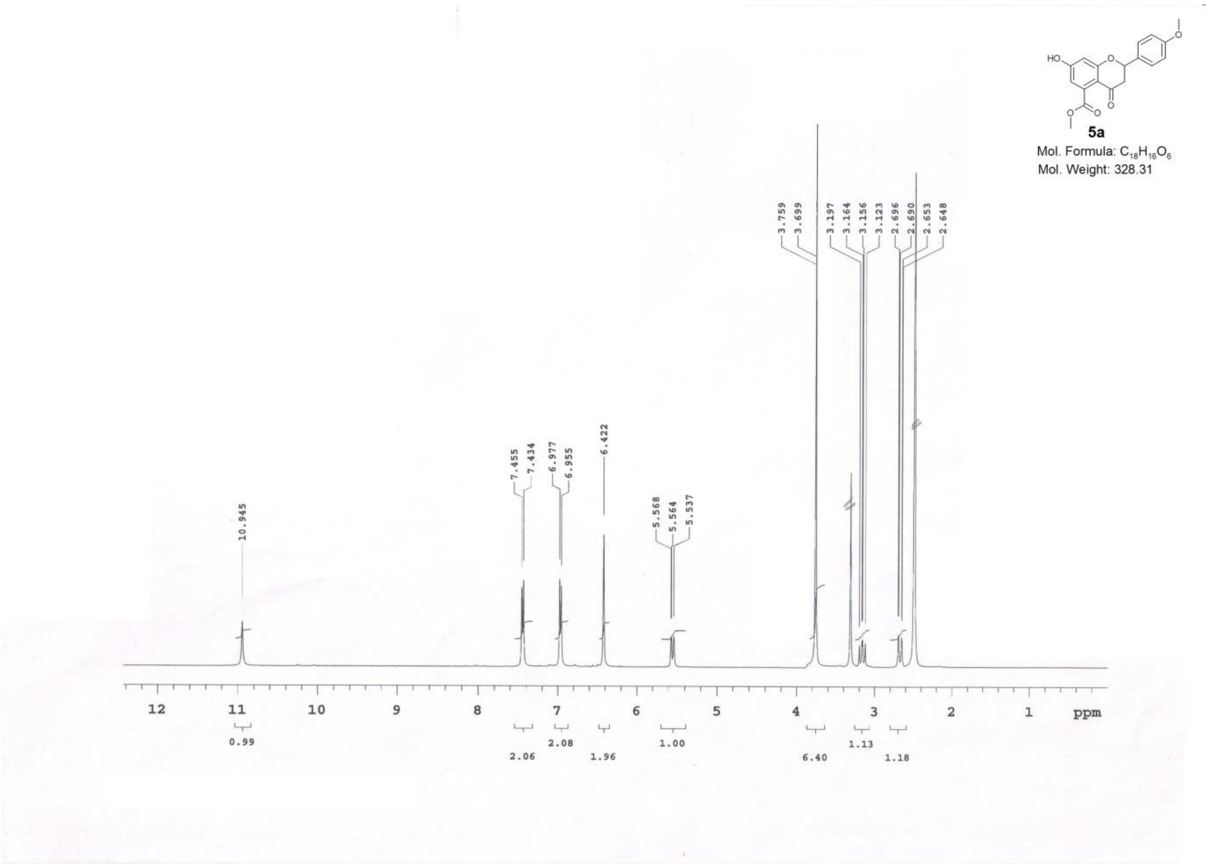
HO
O
O
O
O
O
5a
Mol. Formula: $C_{18}H_{16}O_6$
Mol. Weight: 328.31
10.945
7.455
7.434
6.977
6.955
6.422
5.568
5.564
5.537
3.759
3.699
3.197
3.164
3.156
3.123
2.696
2.690
2.653
2.648
12 11 10 9 8 7 6 5 4 3 2 1 ppm
0.99
2.06
2.08
1.96
1.00
6.40
1.13
1.18

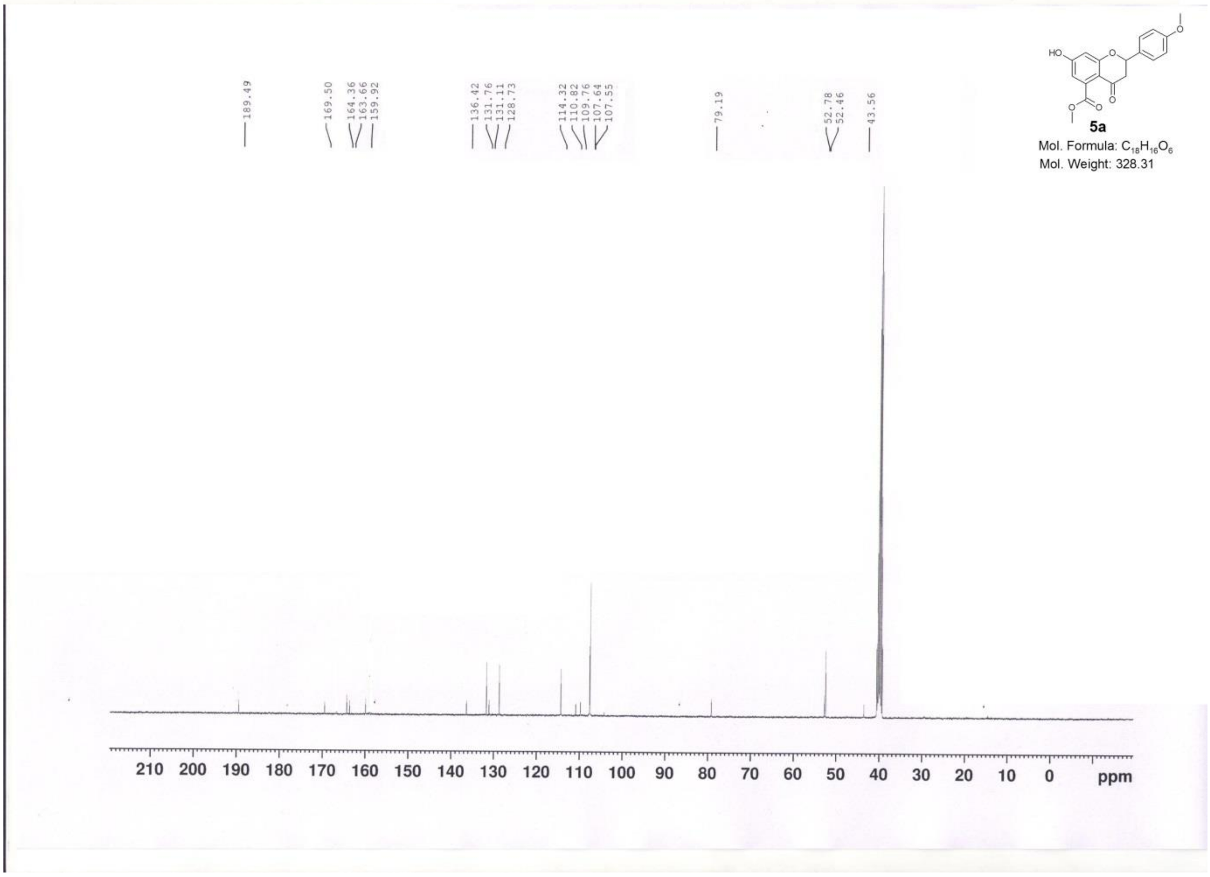
HO
O
O
O
O
O
5a
Mol. Formula: $C_{18}H_{16}O_6$
Mol. Weight: 328.31
189.49
169.50
164.36
163.66
159.92
136.42
131.76
131.11
128.73
114.32
110.82
109.76
107.64
107.55
79.19
52.78
52.46
43.56
210 200 190 180 170 160 150 140 130 120 110 100 90 80 70 60 50 40 30 20 10 0 ppm

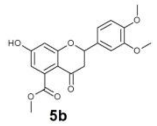

5b

Mol. Formula: $C_{19}H_{18}O_7$
Mol. Weight.: 358.34

LCMS REPORT

Column : X-Select CSH C18 (3.0*50) mm 2.5u
Mobile Phase: A: 0.05% Formic acid in water + 5%ACN
B: 0.05% Formic acid in Acetonitrile
Inj Volume; 2.0μL,
Flow Rate: 1.2 mL/minute
Gradient program:
0% B to 98 % B in 2.0 minute, hold till 3.0 min, at 3.2 min B conc is 0 % up to 4.0 min

Chromatogram

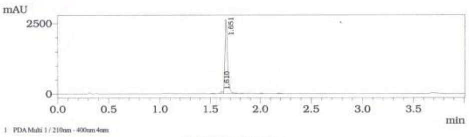

MS Chromatogram

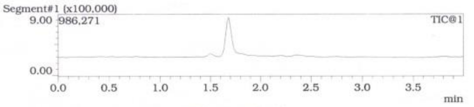

MS Spectrum Graph

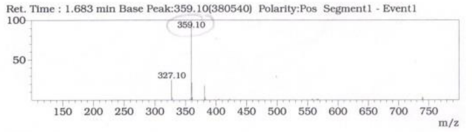

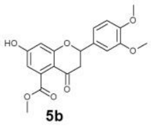

5b

Mol. Formula: $C_{19}H_{18}O_7$
Mol. Weight.: 358.34

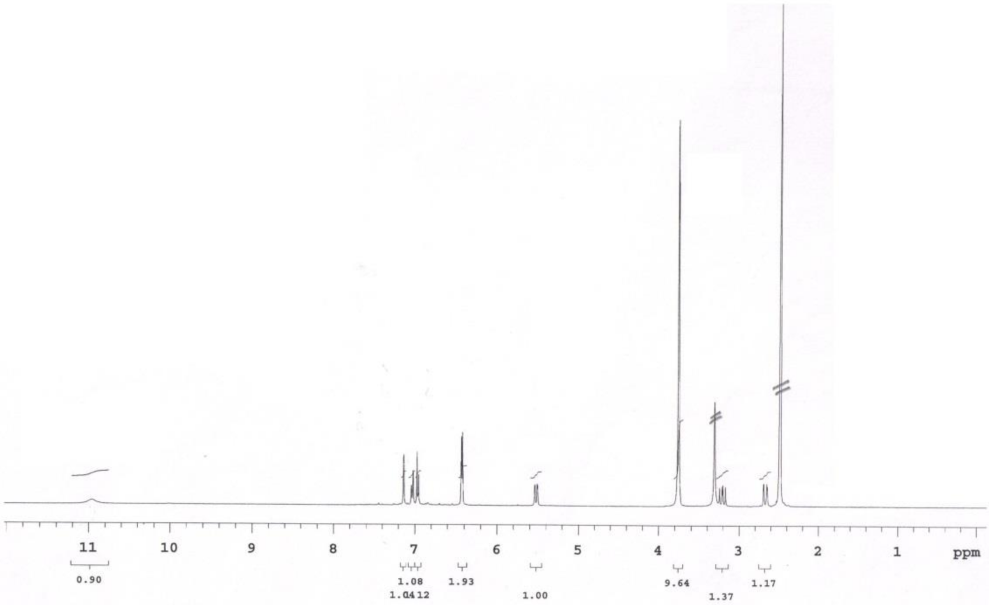

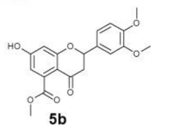

5b

Mol. Formula: $C_{19}H_{18}O_7$

Mol. Weight.: 358.34

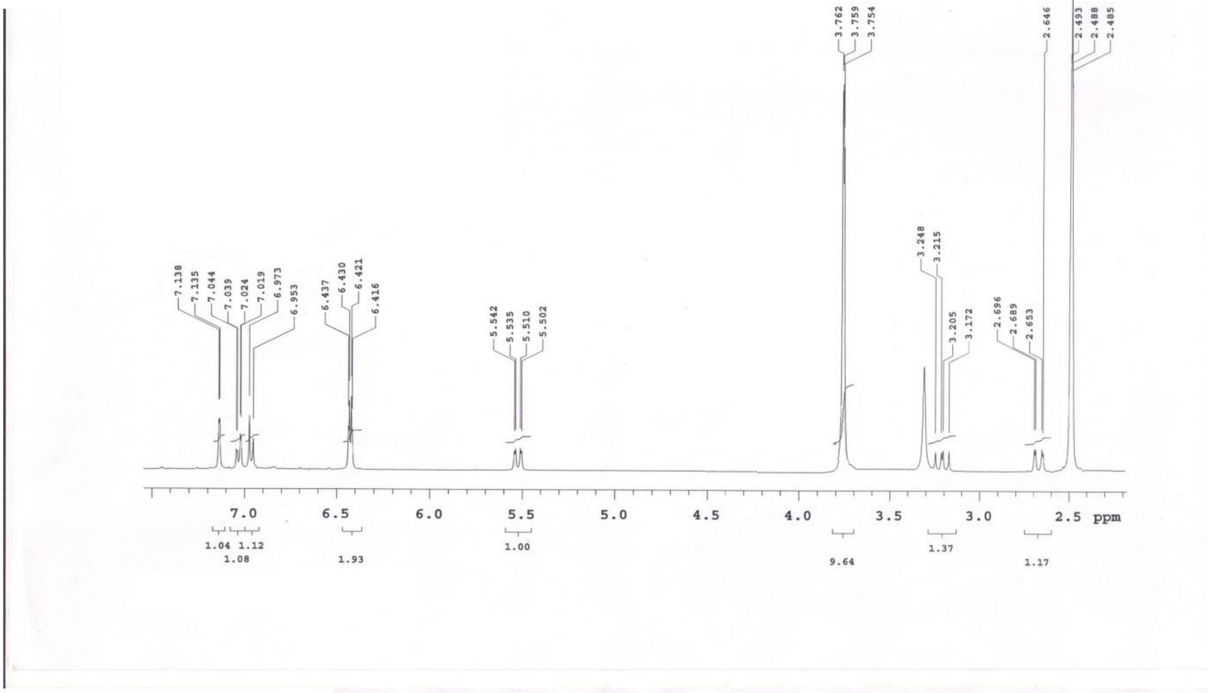

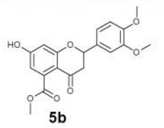

5b

Mol. Formula: $C_{19}H_{18}O_7$

Mol. Weight.: 358.34

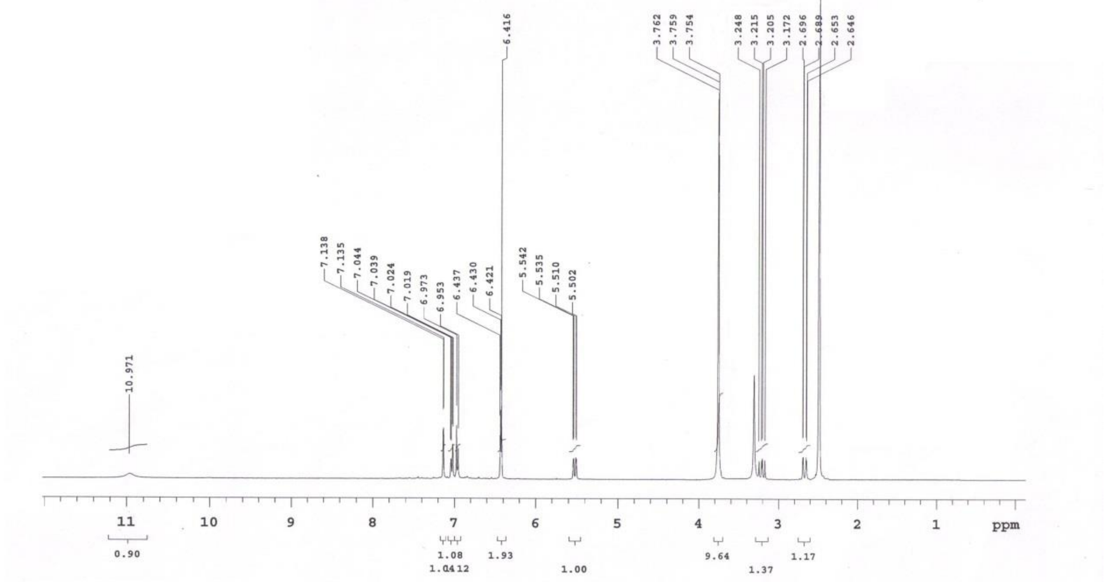

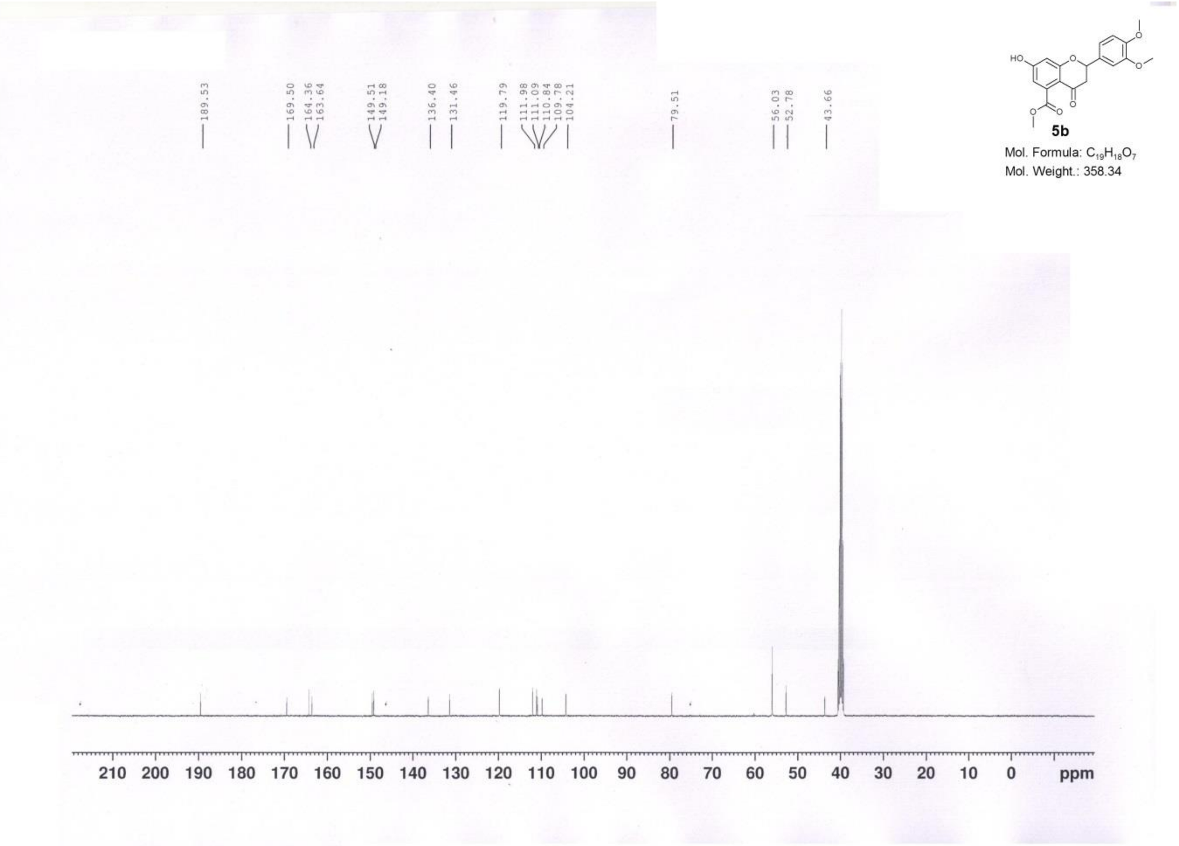

189.53
169.50
164.36
163.64
149.51
149.18
136.40
131.46
119.79
111.98
111.09
110.84
109.78
104.21
79.51
56.03
52.78
43.66
HO
5b
Mol. Formula: $C_{19}H_{18}O_7$
Mol. Weight.: 358.34
210 200 190 180 170 160 150 140 130 120 110 100 90 80 70 60 50 40 30 20 10 0 ppm

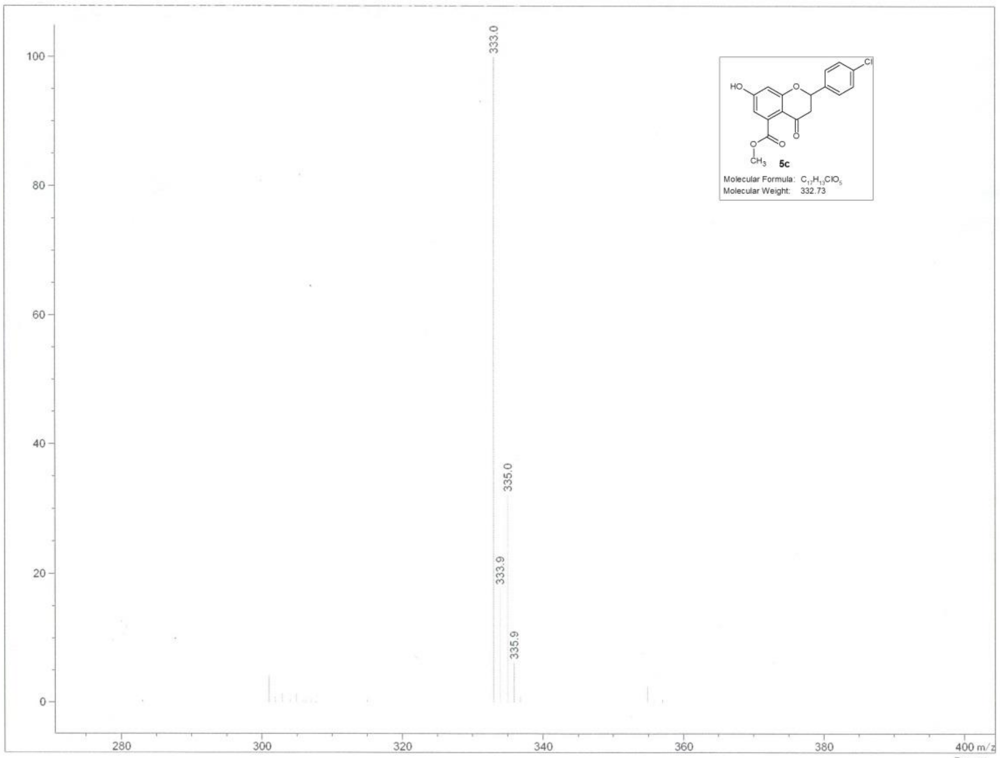

333.0
335.0
333.9
335.9
100
80
60
40
20
0
HO
Cl
CH_3
5c
Molecular Formula:
Molecular Weight: 332.73
280
300
320
340
360
380
400 m/z

Page 1 of 1

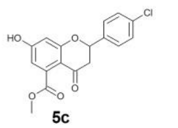

5c

Mol. Formula: $C_{17}H_{13}ClO_5$

Mol. Weight: 332.73

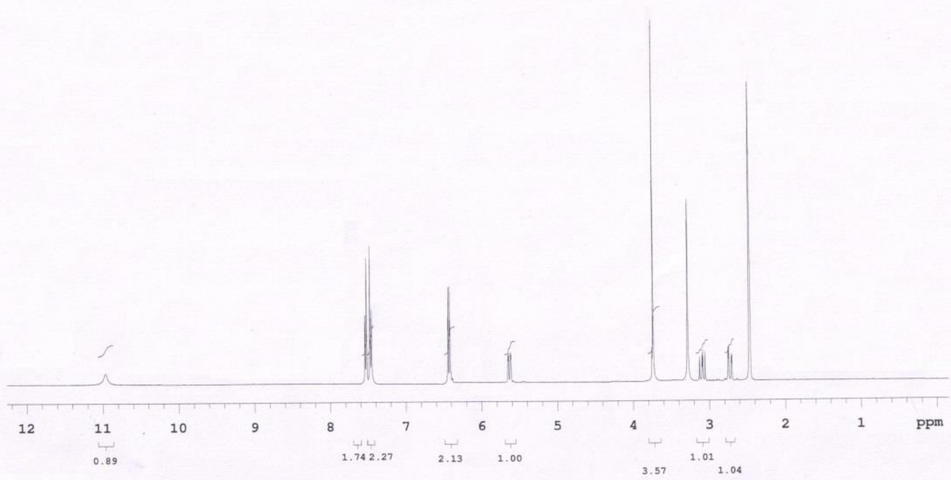

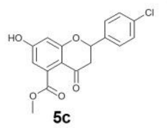

5c

Mol. Formula: $C_{17}H_{13}ClO_5$

Mol. Weight: 332.73

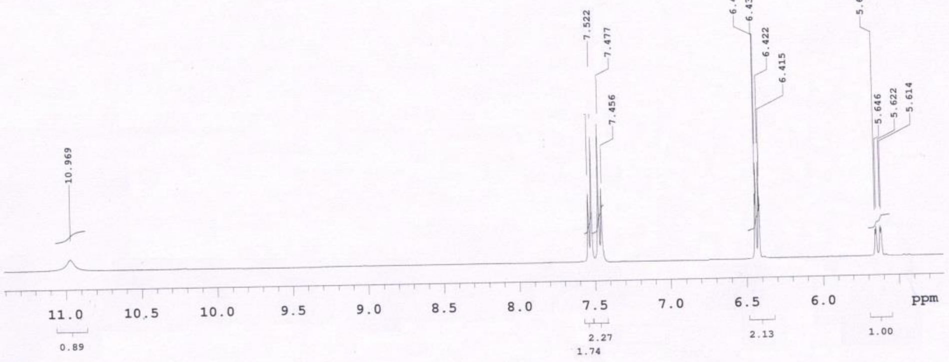

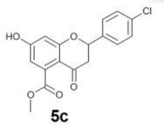

5c

Mol. Formula: $C_{17}H_{13}ClO_5$
Mol. Weight: 332.73

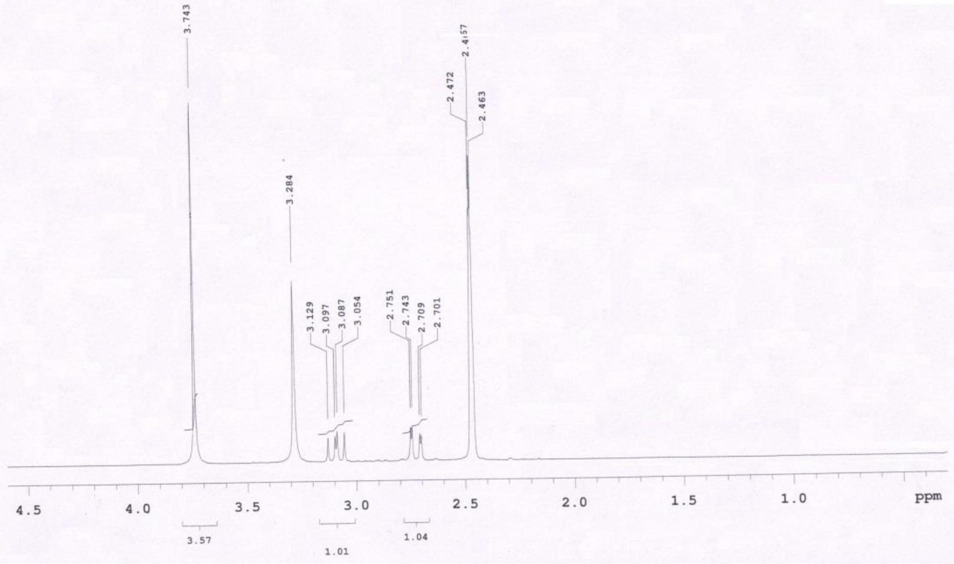

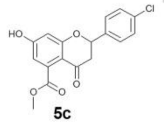

5c

Mol. Formula: $C_{17}H_{13}ClO_5$
Mol. Weight: 332.73

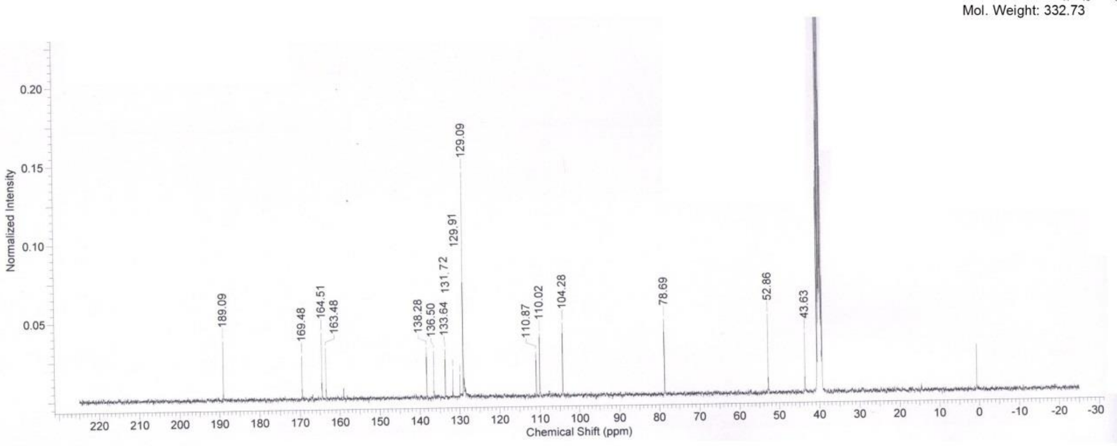

MS Spectrum

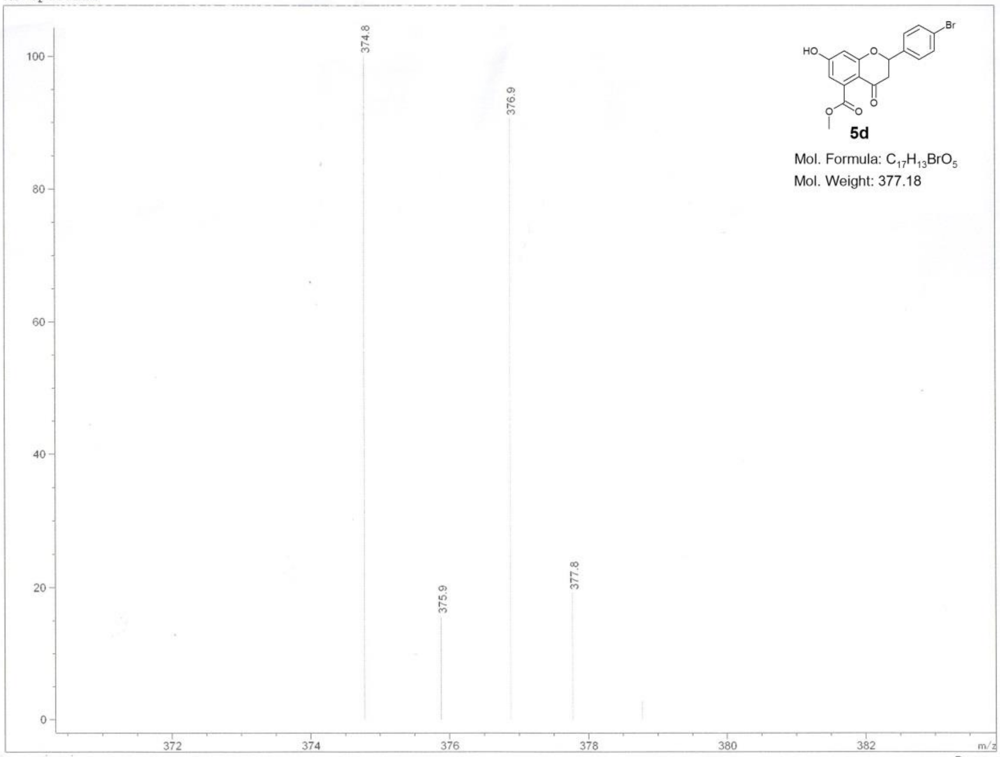
374.8
376.9
375.9
377.8
5d
Mol. Formula: $C_{17}H_{13}BrO_5$
Mol. Weight: 377.18
m/z

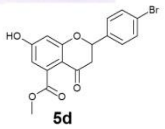

5d

Mol. Formula: $C_{17}H_{13}BrO_5$

Mol. Weight: 377.18

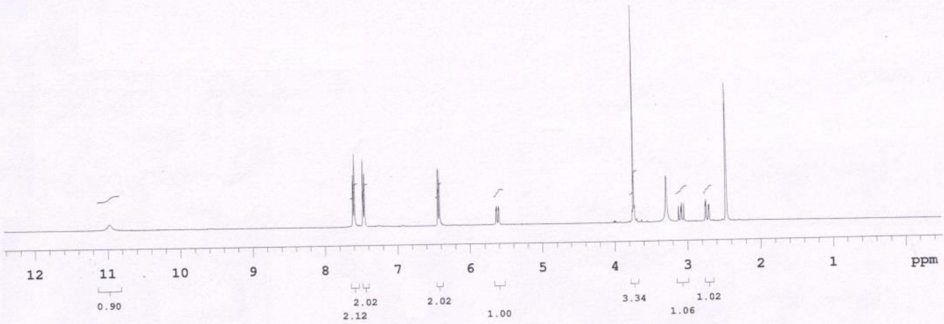

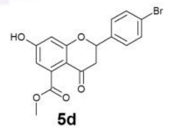

5d

Mol. Formula: $C_{17}H_{13}BrO_5$

Mol. Weight: 377.18

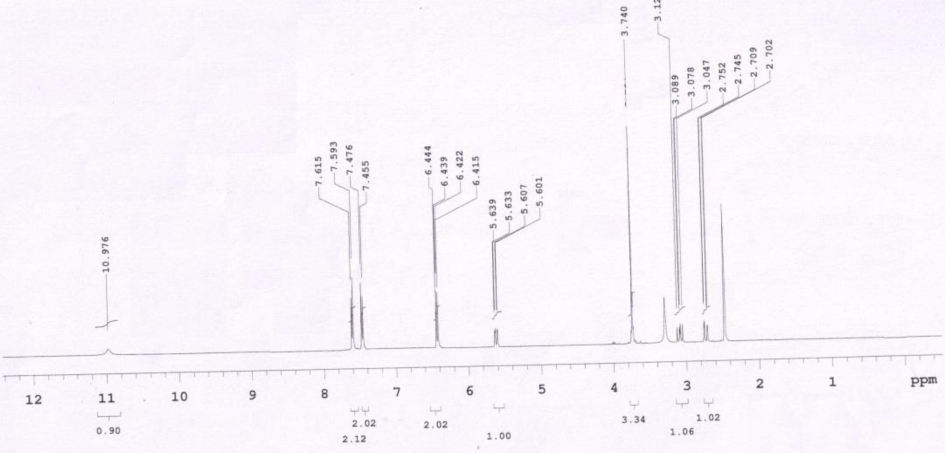

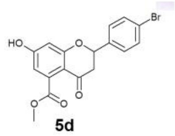

5d

Mol. Formula: $C_{17}H_{13}BrO_5$
Mol. Weight: 377.18

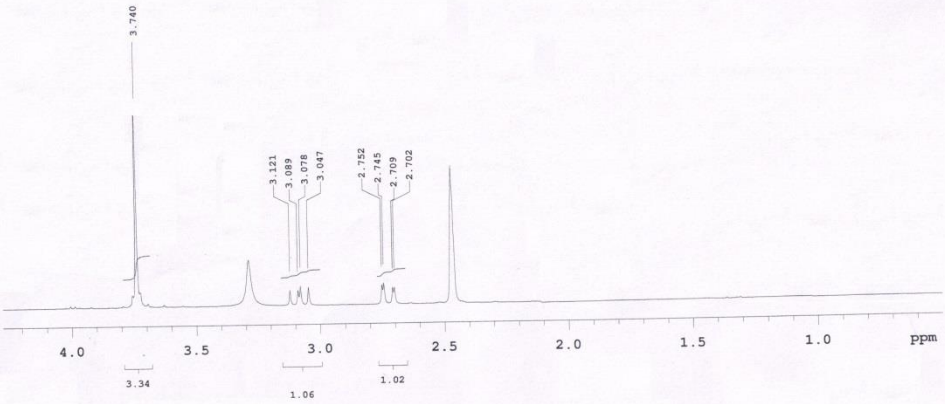

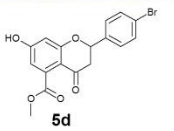

5d

Mol. Formula: $C_{17}H_{13}BrO_5$
Mol. Weight: 377.18

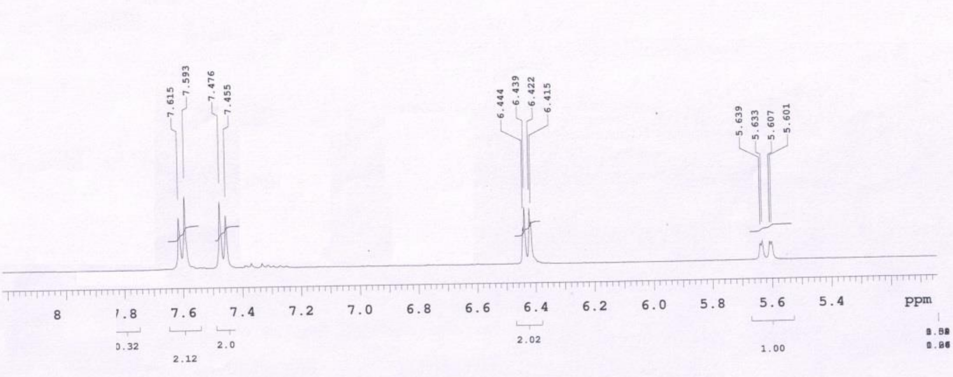

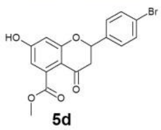

5d

Mol. Formula: $C_{17}H_{13}BrO_5$

Mol. Weight: 377.18

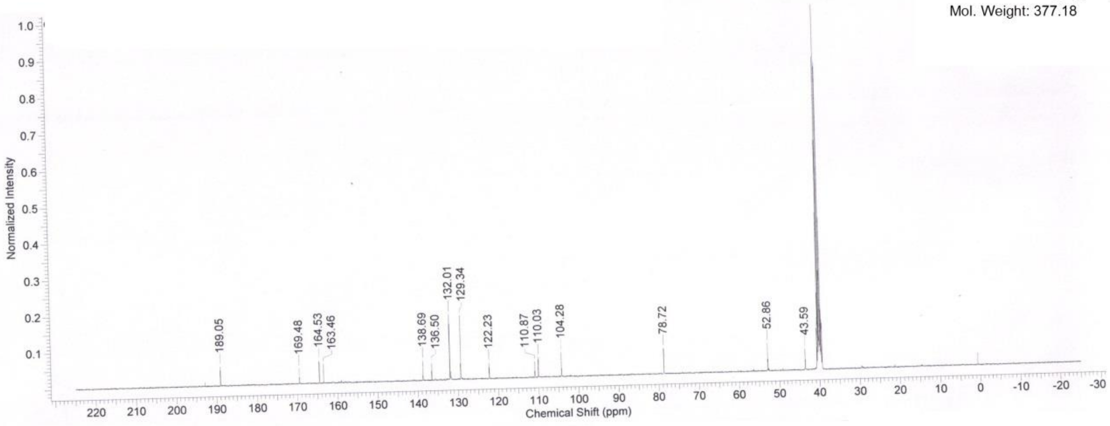

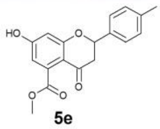

5e

Mol. Formula: $C_{18}H_{16}O_5$

Mol. Weight: 312.31

LCMS REPORT

Column : X-Select CSH (3.0*50) mm 2.5u
Mobile Phase: A: 0.05% Formic acid in water+5%ACN
B: 0.05% Fornic acid in ACN
Inj Volume: 2.0µL
Flow Rate: 1.5. mL/minute
Gradient program:
0% B to 98 % B in 2.0 minute, Hold till 3.0 min,At 3.2 min B conc is 0 % up to 4.0 min

Chromatogram

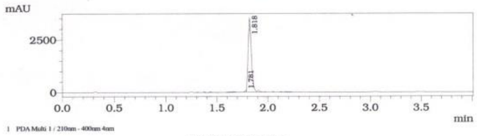

1 PDA Multi 1 / 210nm - 400nm 4nm

MS Chromatogram

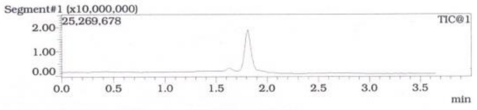

MS Spectrum Graph

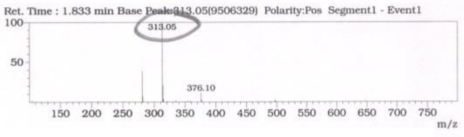

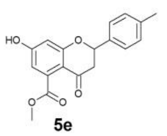

5e

Mol. Formula: $C_{18}H_{16}O_5$

Mol. Weight: 312.31

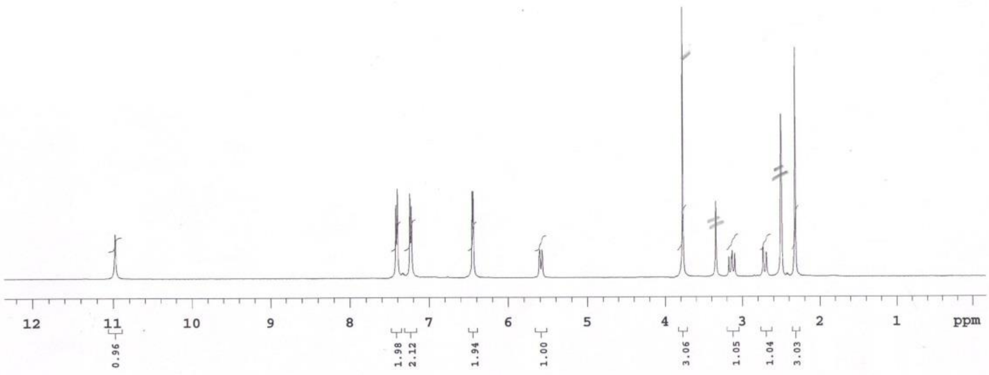

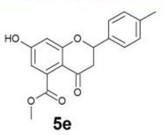

5e

Mol. Formula: $C_{18}H_{16}O_5$

Mol. Weight: 312.31

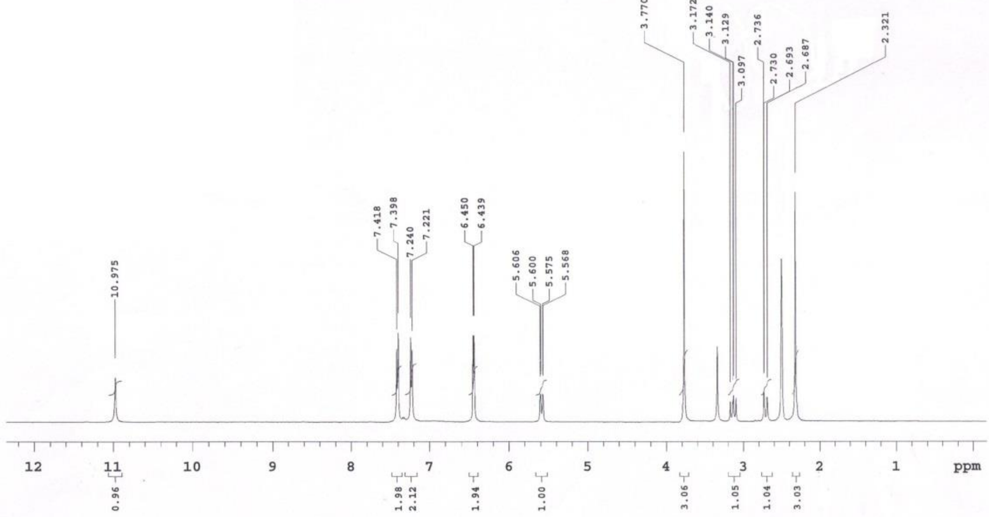

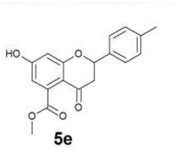

5e

Mol. Formula: $C_{18}H_{16}O_5$
Mol. Weight: 312.31

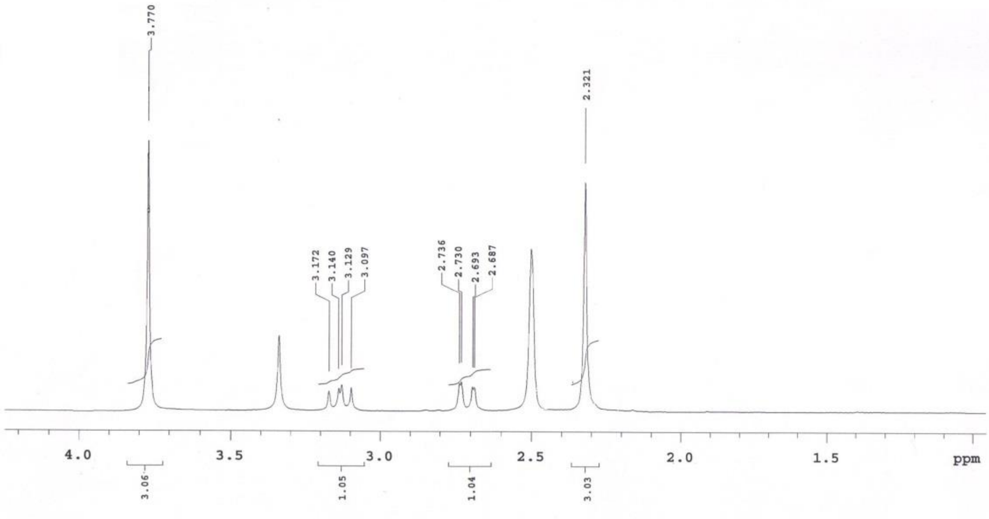

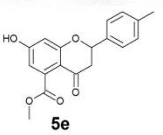

5e

Mol. Formula: $C_{18}H_{16}O_5$
Mol. Weight: 312.31

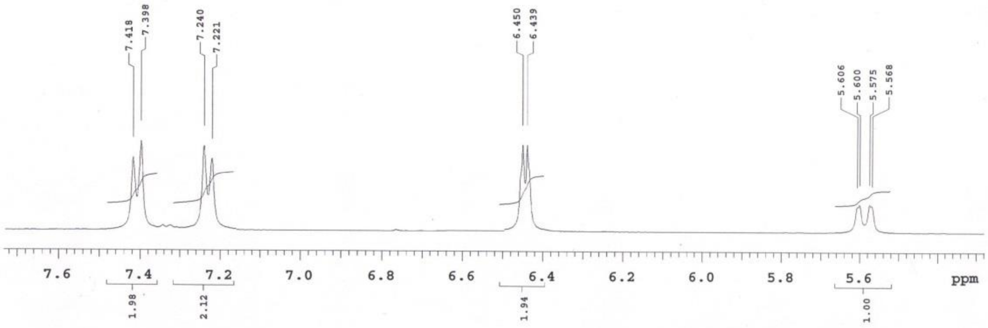

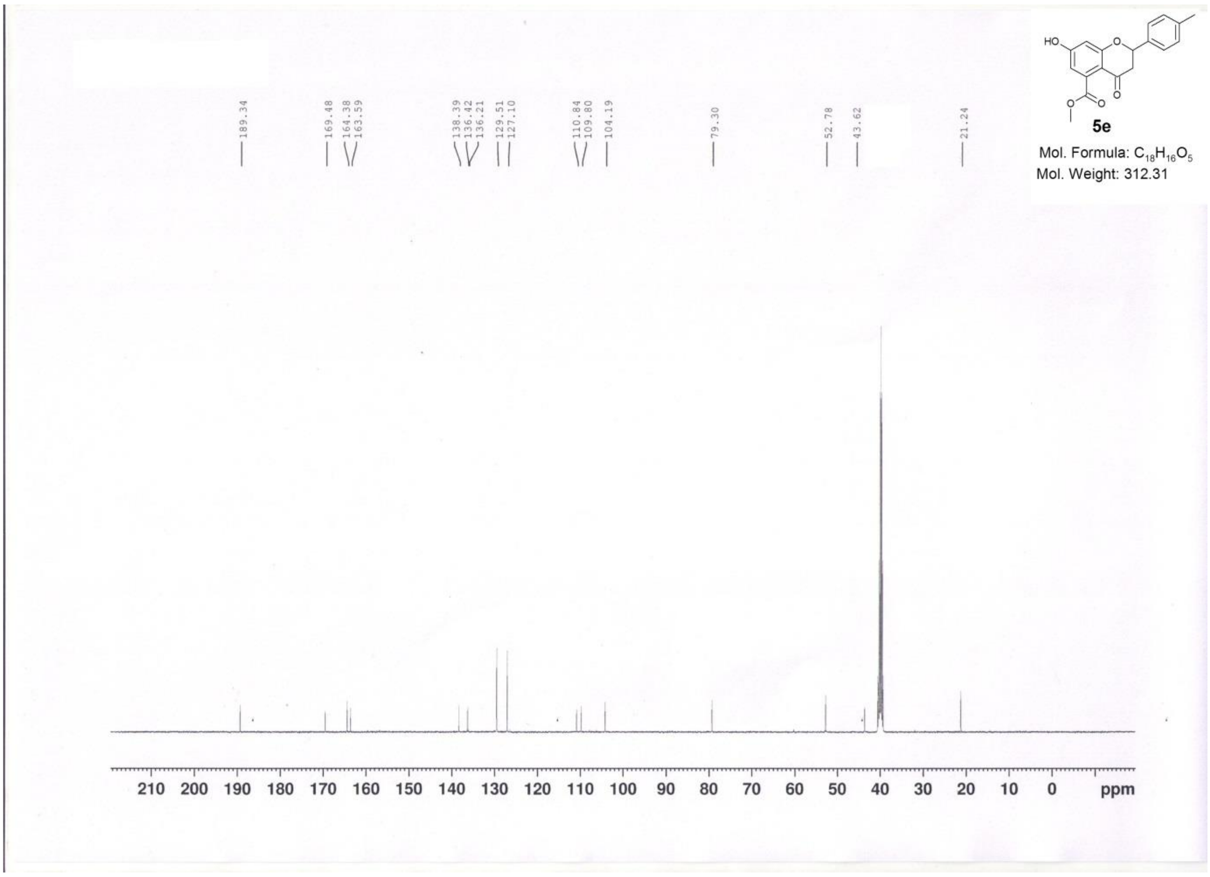
189.34
169.48
164.38
163.59
138.39
136.42
136.21
129.51
127.10
110.84
109.80
104.19
79.30
52.78
43.62
21.24
HO
O
O
O
O
5e
Mol. Formula: $C_{18}H_{16}O_5$
Mol. Weight: 312.31
210 200 190 180 170 160 150 140 130 120 110 100 90 80 70 60 50 40 30 20 10 0 ppm

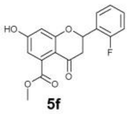

5f

Mol. Formula: $C_{17}H_{13}FO_5$

Mol. Weight: 316.28

LCMS REPORT

Column : X-Select CSH (3.0*50) mm 2.5u
Mobile Phase: A: 0.05% Fornic acid in water+5%ACN
B: 0.05% Fornic acid in ACN
Inj Volume; 2.0µL,
Flow Rate: 1.5. mL/minute
Gradient program:
0% B to 98 % B in 2.0 minute, Hold till 3.0 min,At 3.2 min B conc is 0 % up to 4.0 min

Chromatogram

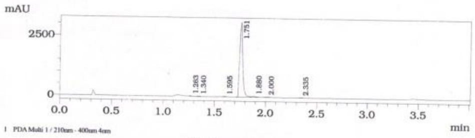

MS Chromatogram

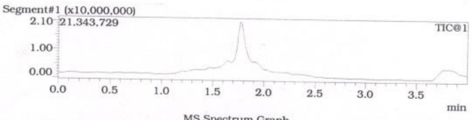

MS Spectrum Graph

Ret. Time : 1.767 min Base Peak:317.05(9035755) Polarity:Pos Segment1 - Event1

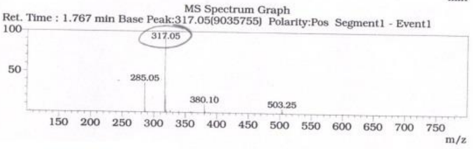

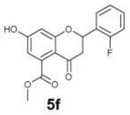

5f

Mol. Formula: $C_{17}H_{13}FO_5$

Mol. Weight: 316.28

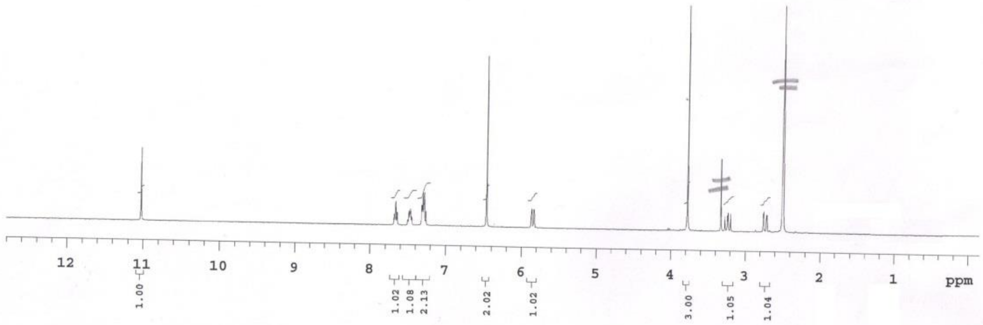

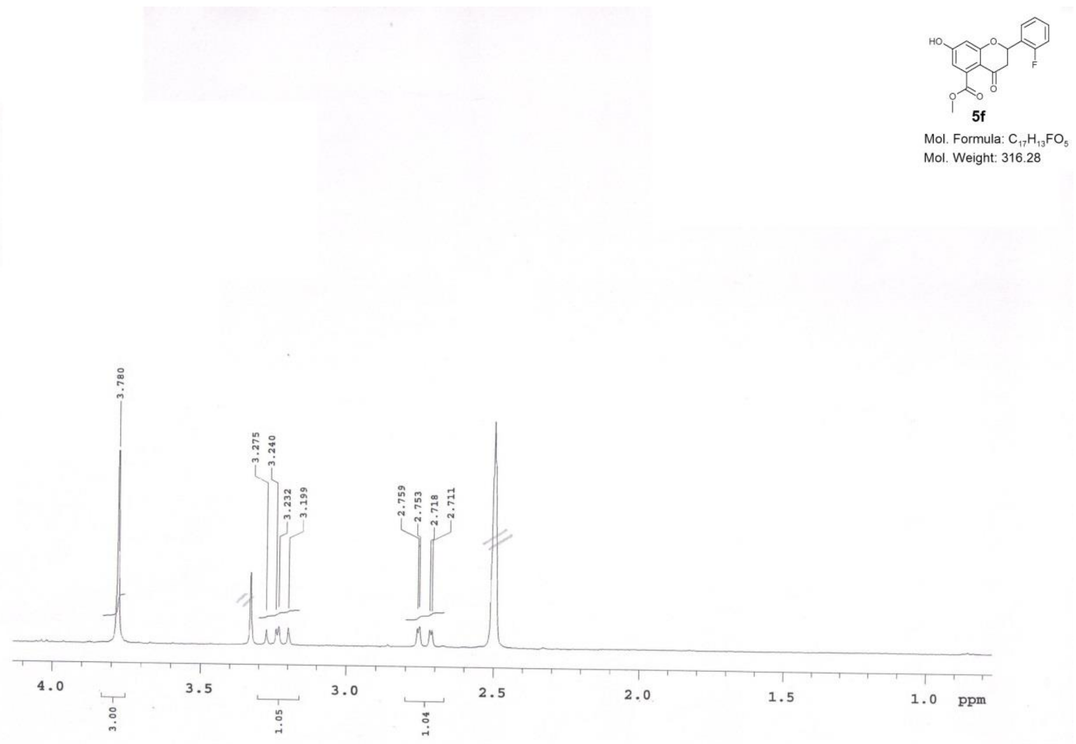
HO
O
F
O
O
O
5f
Mol. Formula: $C_{17}H_{13}FO_5$
Mol. Weight: 316.28
ppm

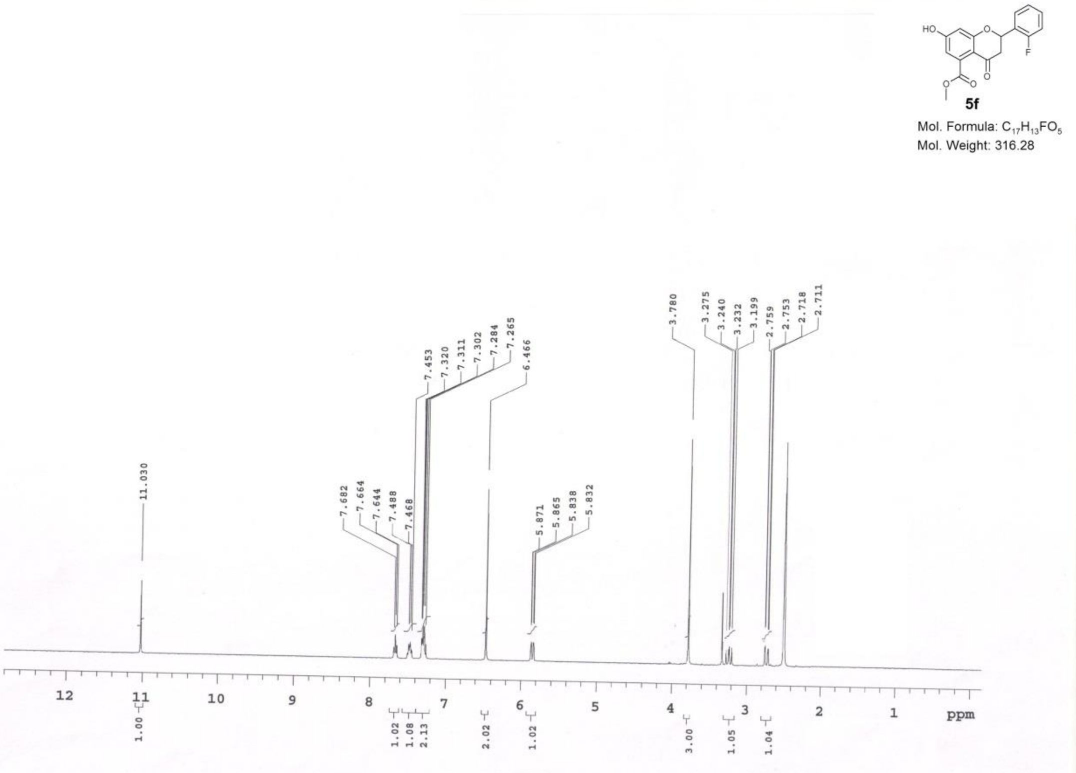
HO
O
F
O
O
O
5f
Mol. Formula: $C_{17}H_{13}FO_5$
Mol. Weight: 316.28
ppm

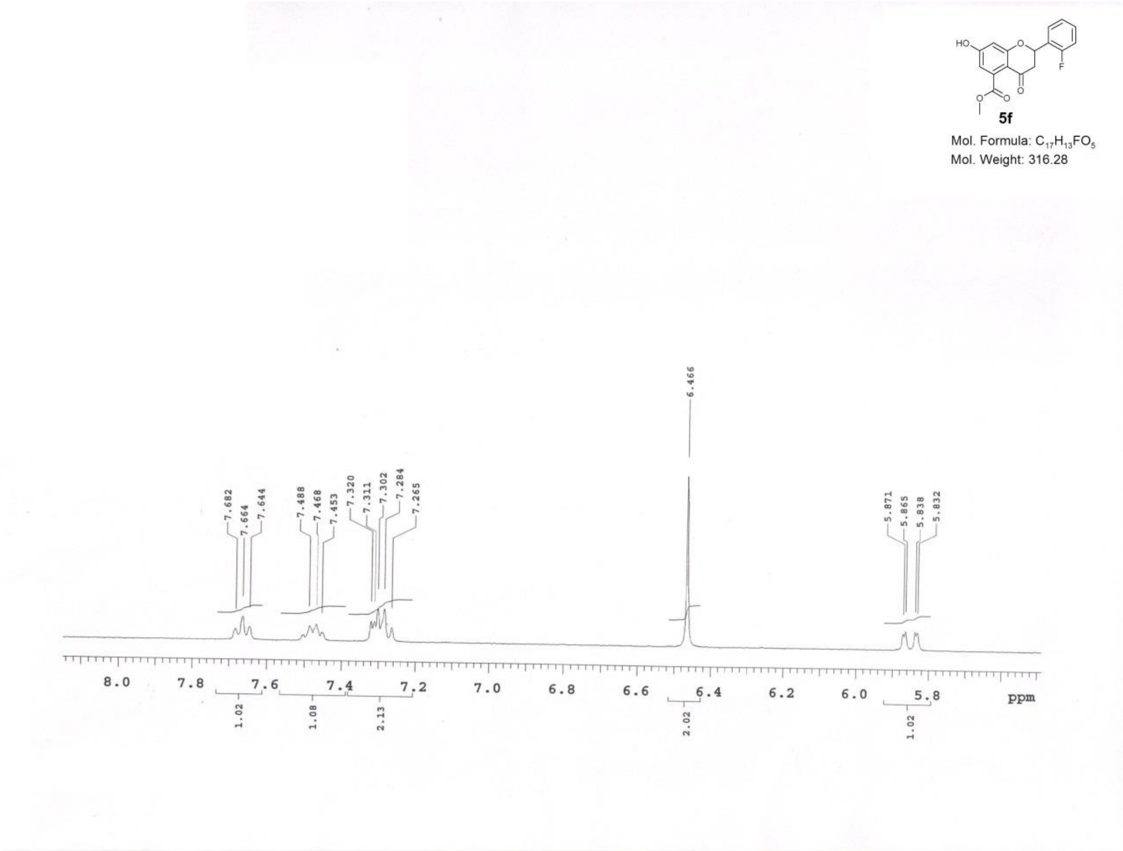
HO
O
F
O
O
O
5f
Mol. Formula: $C_{17}H_{13}FO_5$
Mol. Weight: 316.28
7.682
7.664
7.644
7.488
7.468
7.453
7.320
7.311
7.302
7.284
7.265
6.466
5.871
5.865
5.838
5.832
8.0 7.8 7.6 7.4 7.2 7.0 6.8 6.6 6.4 6.2 6.0 5.8 ppm
1.02
1.08
2.13
2.02
1.02

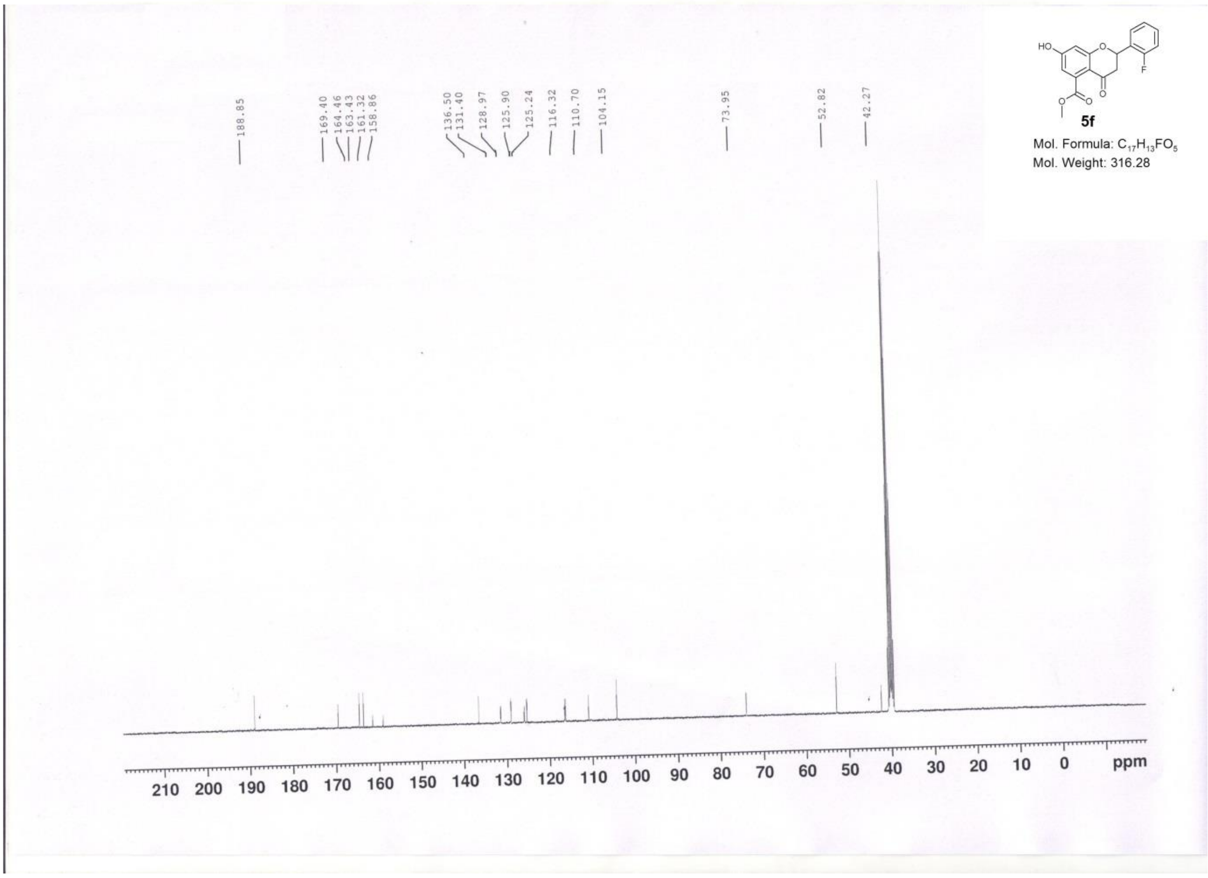
HO
O
F
O
O
O
5f
Mol. Formula: $C_{17}H_{13}FO_5$
Mol. Weight: 316.28
188.85
169.40
164.46
163.43
161.32
158.86
136.50
131.40
128.97
125.90
125.24
116.32
110.70
104.15
73.95
52.82
42.27
210 200 190 180 170 160 150 140 130 120 110 100 90 80 70 60 50 40 30 20 10 0 ppm

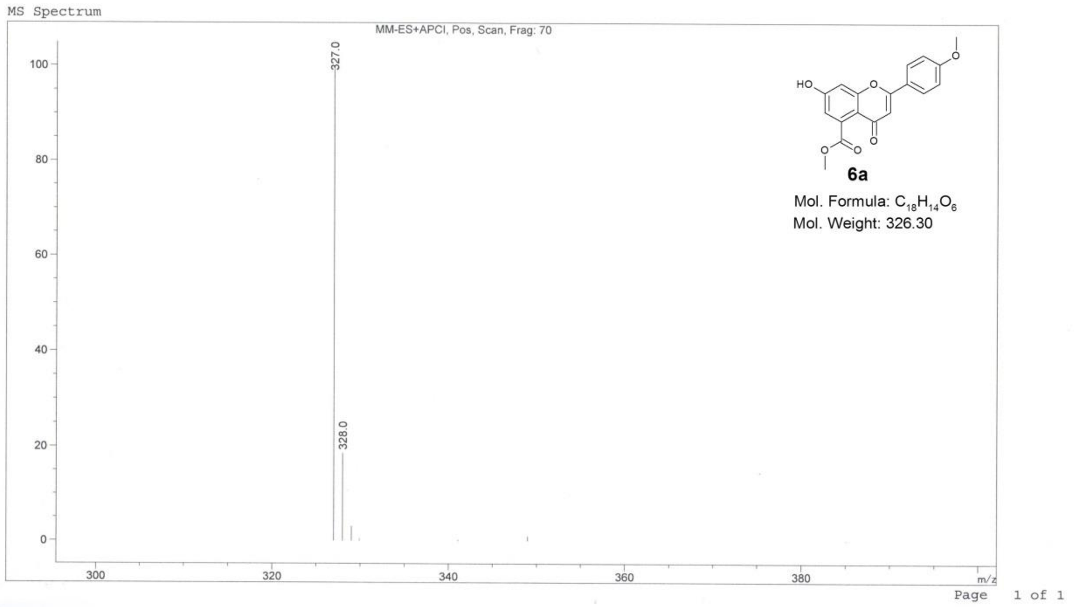
MS Spectrum
MM-ES+APCI, Pos, Scan, Frag: 70
327.0
328.0
100
80
60
40
20
0
300
320
340
360
380
m/z
HO
O
O
O
O
O
6a
Mol. Formula: $C_{18}H_{14}O_6$
Mol. Weight: 326.30
Page 1 of 1

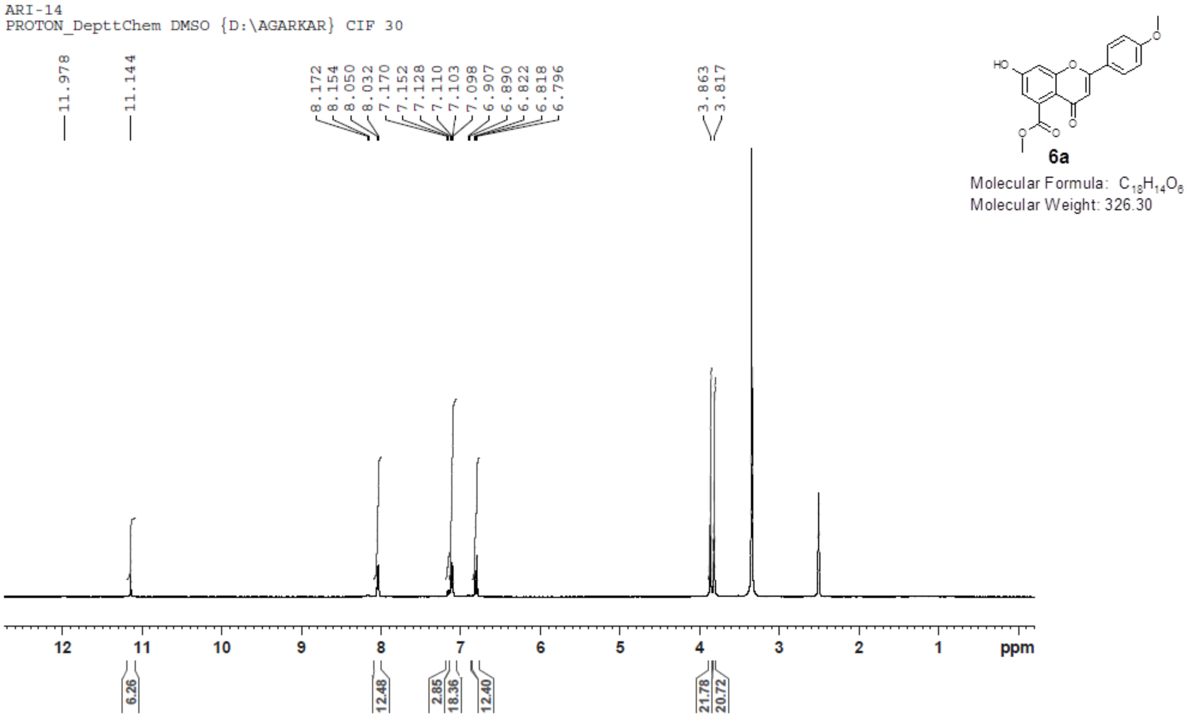
ARI-14
PROTON_DepttChem DMSO {D:\AGARKAR} CIF 30
11.978
11.144
8.172
8.154
8.050
8.032
7.170
7.152
7.128
7.110
7.103
7.098
6.907
6.890
6.822
6.818
6.796
3.863
3.817
HO
O
O
O
O
O
6a
Molecular Formula: $C_{18}H_{14}O_6$
Molecular Weight: 326.30
12
11
10
9
8
7
6
5
4
3
2
1
ppm
6.26
12.48
2.85
18.36
12.40
21.78
20.72

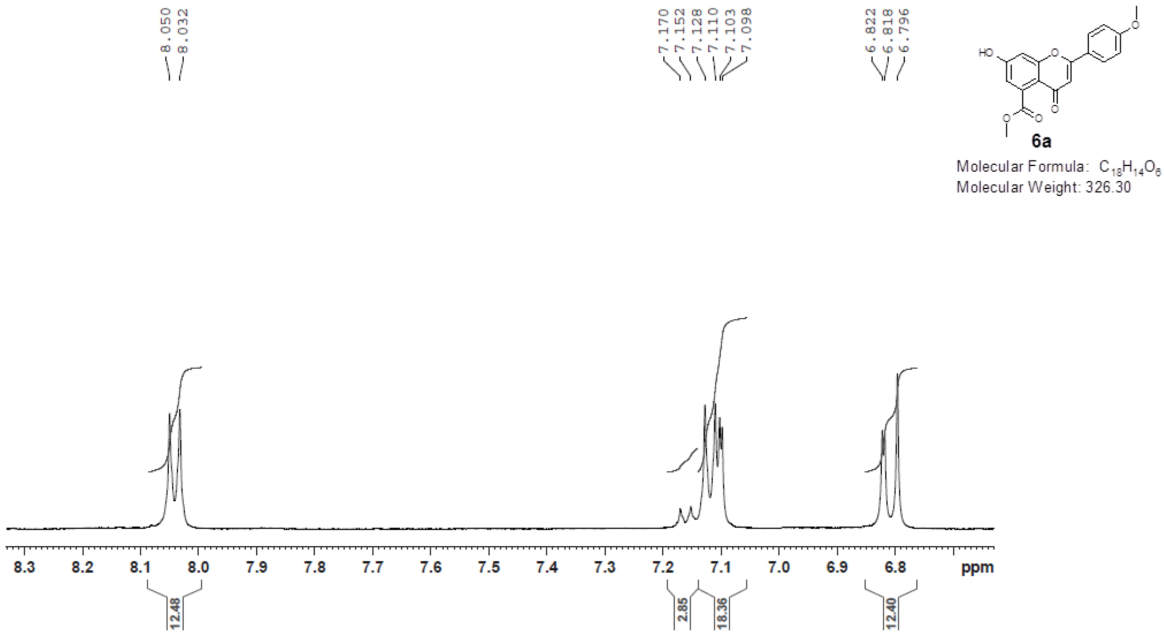
8.050
8.032
7.170
7.152
7.128
7.110
7.103
7.098
6.822
6.818
6.796
HO
O
O
O
O
O
6a
Molecular Formula: C18H14O6
Molecular Weight: 326.30
8.3
8.2
8.1
8.0
7.9
7.8
7.7
7.6
7.5
7.4
7.3
7.2
7.1
7.0
6.9
6.8
ppm
12.48
2.85
18.36
12.40

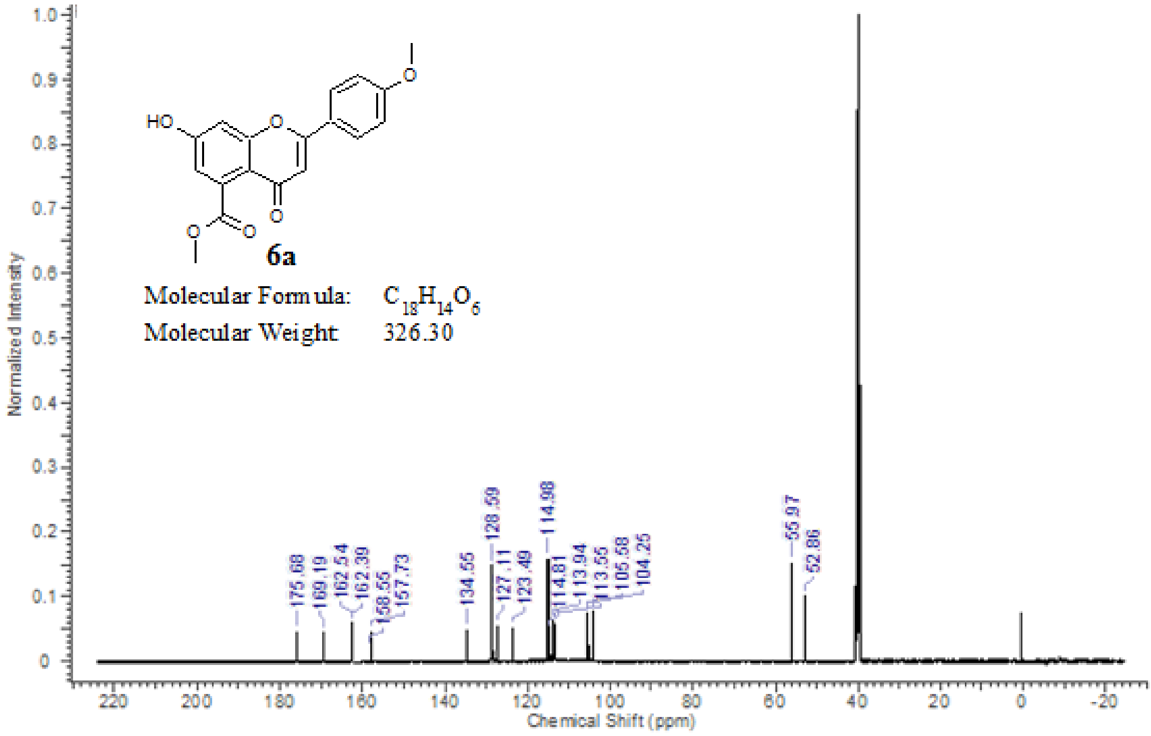
HO
O
O
O
O
O
6a
Molecular Formula: C18H14O6
Molecular Weight 326.30
Normalized Intensity
Chemical Shift (ppm)
175.68
169.19
162.54
162.39
158.55
157.73
134.55
128.59
127.11
123.49
114.98
114.81
113.94
113.55
105.58
104.25
55.97
52.86

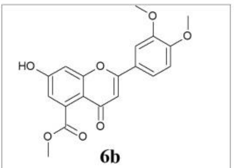

6b

Molecular Formula: $C_{19}H_{16}O_7$

Molecular Weight: 356.32

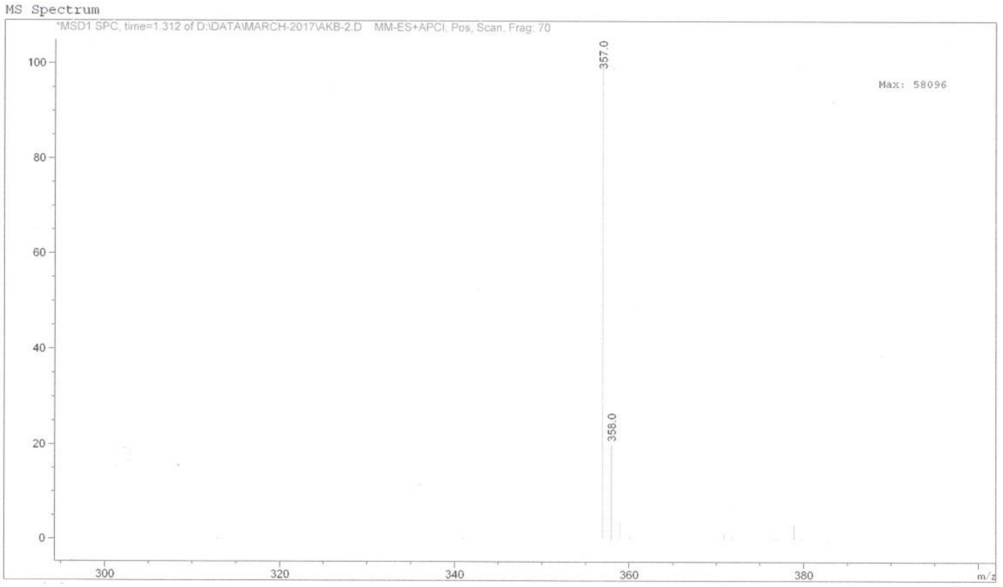

Page 1 of 1

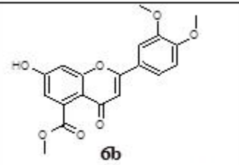

6b

Molecular Formula: $C_{19}H_{16}O_7$

Molecular Weight: 356.32

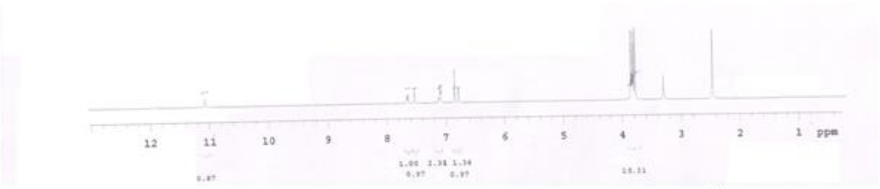

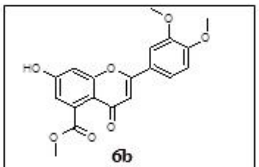

6b

Molecular Formula: $C_{19}H_{16}O_7$

Molecular Weight: 356.32

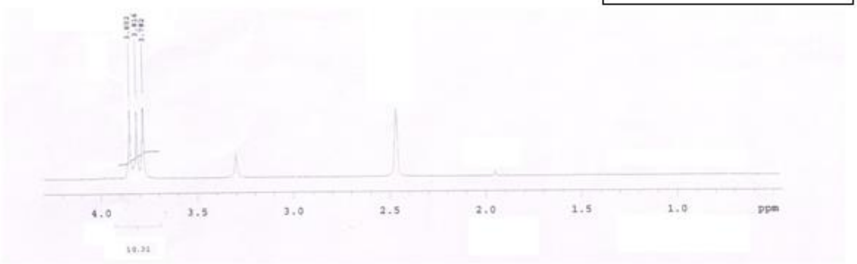

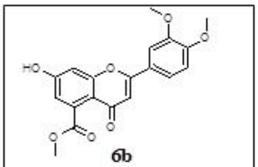

6b

Molecular Formula: $C_{19}H_{16}O_7$

Molecular Weight: 356.32

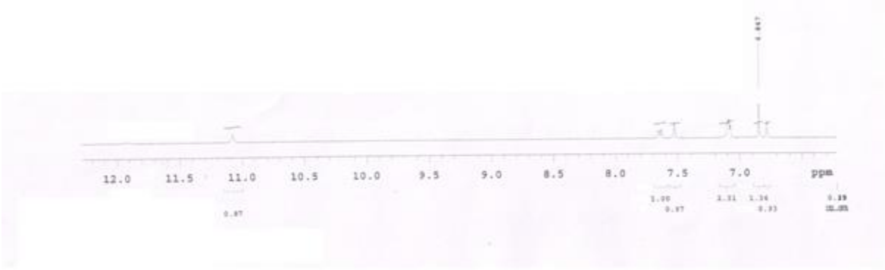

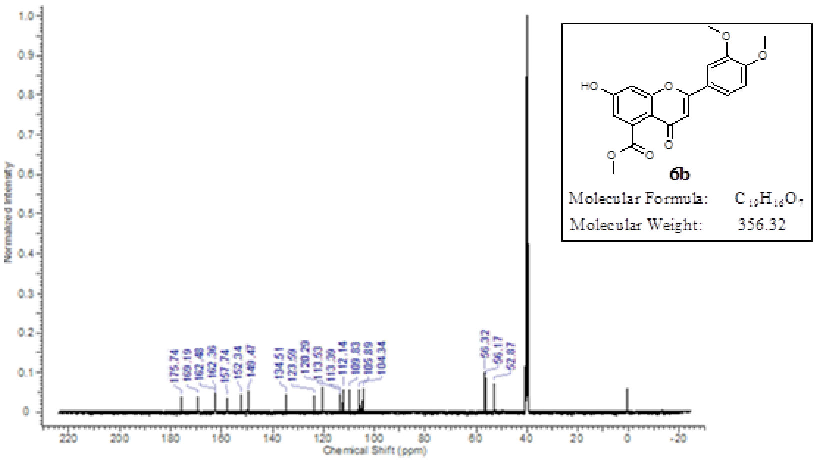

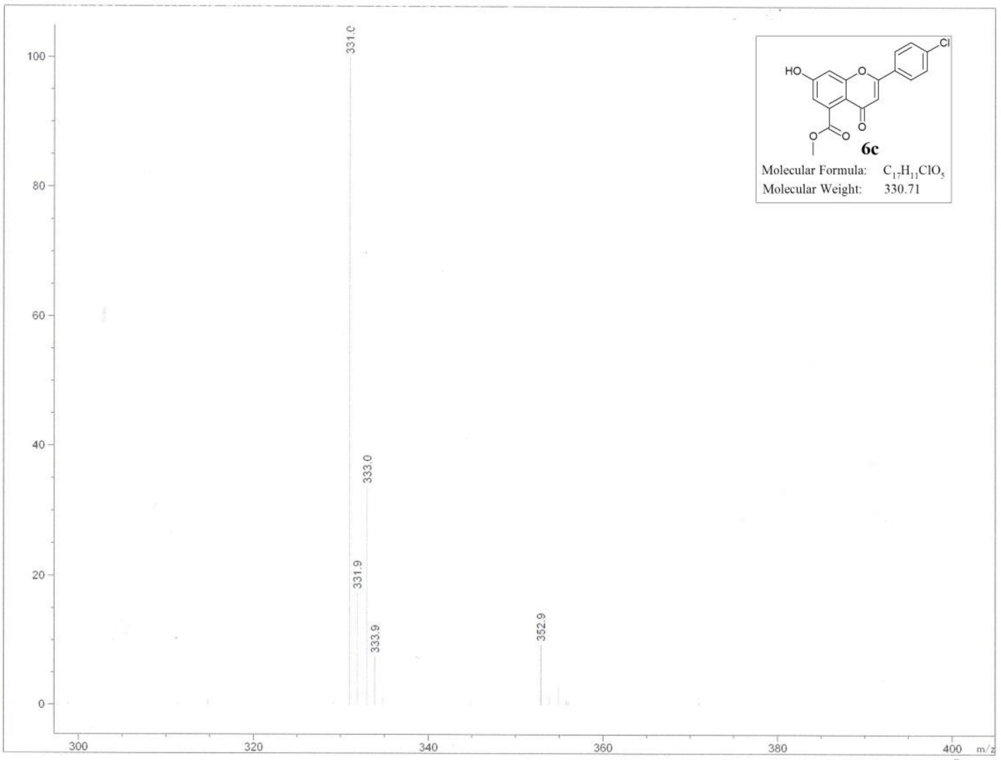

Page 1 of 1

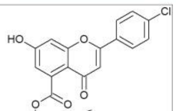

6c

Molecular Formula: $C_{17}H_{11}ClO_5$
Molecular Weight: 330.71

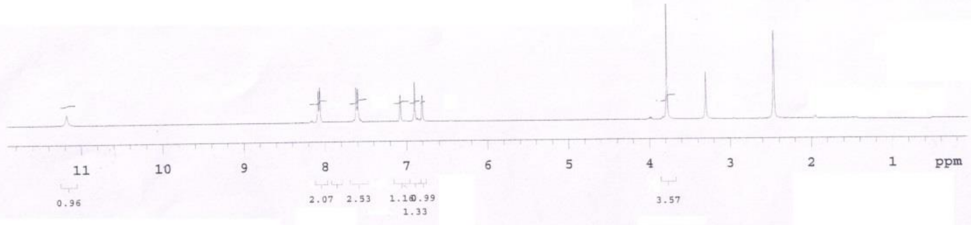

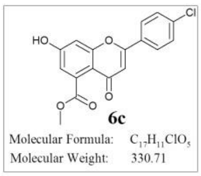

6c

Molecular Formula: $C_{17}H_{11}ClO_5$

Molecular Weight: 330.71

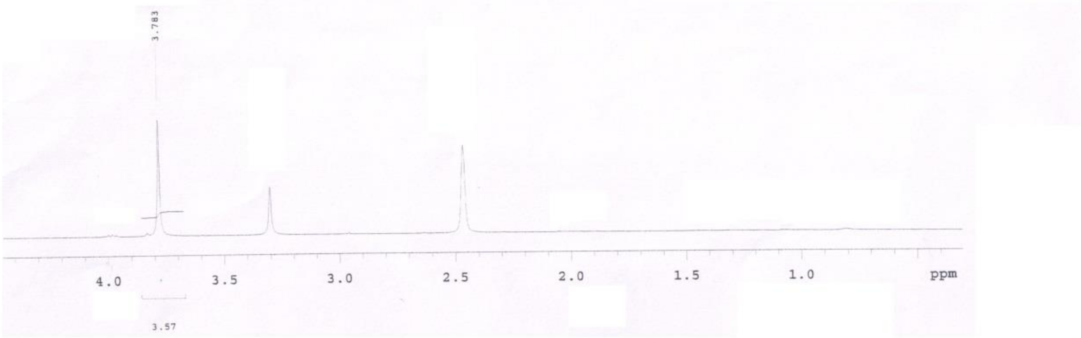

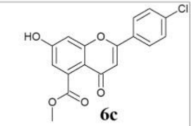

6c

Molecular Formula: $C_{17}H_{11}ClO_5$

Molecular Weight: 330.71

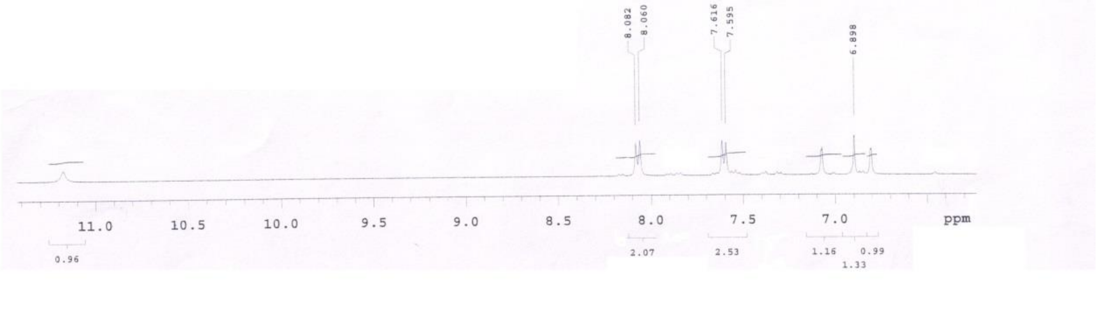

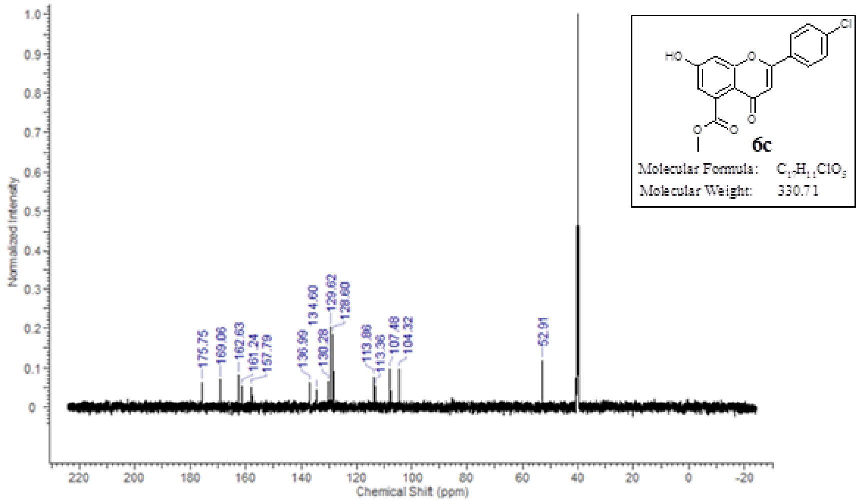
175.75
169.06
162.63
161.24
157.79
136.99
134.60
130.28
129.62
128.60
113.86
113.36
107.48
104.32
52.91
Normalized Intensity
Chemical Shift (ppm)
HO
Cl
6c
Molecular Formula: C17H11ClO5
Molecular Weight: 330.71

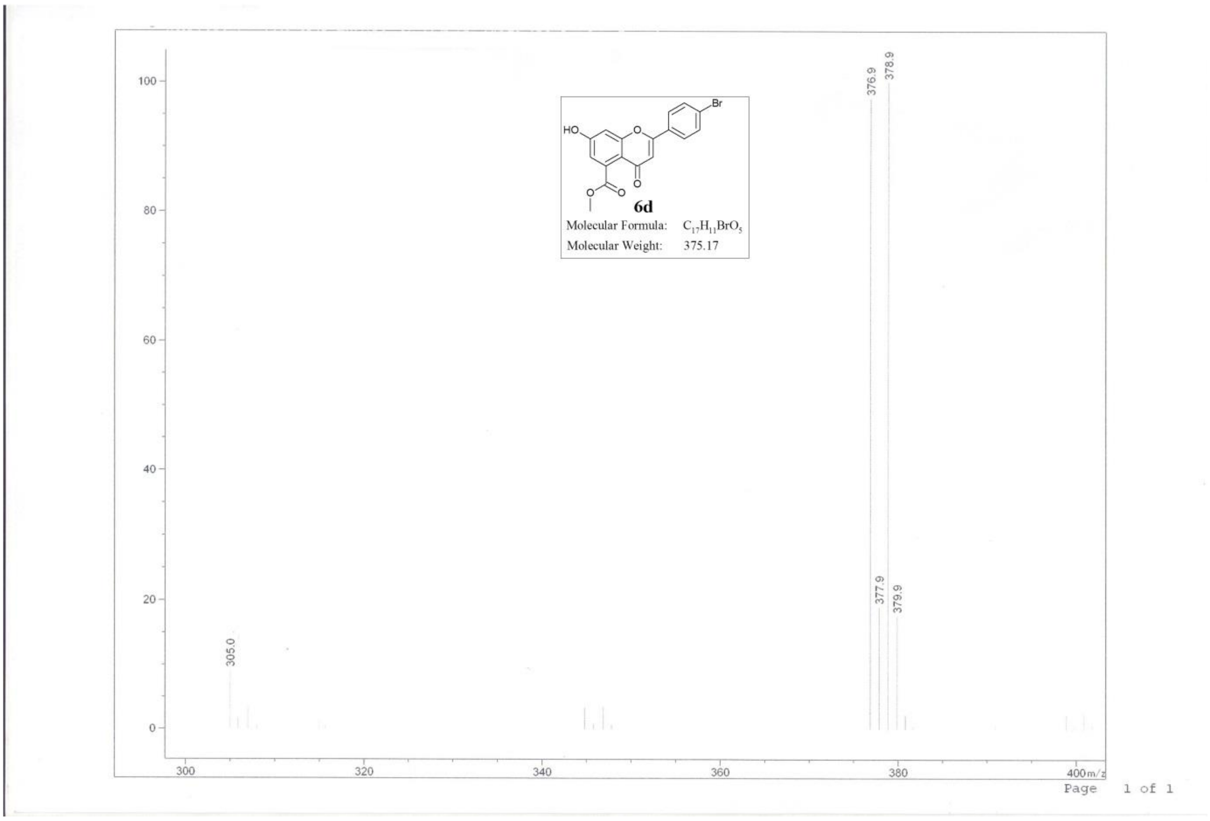
376.9
378.9
377.9
379.9
305.0
HO
Br
6d
Molecular Formula: C17H11BrO5
Molecular Weight: 375.17
m/z
Page 1 of 1

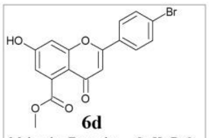

6d

Molecular Formula: $C_{17}H_{11}BrO_5$

Molecular Weight: 375.17

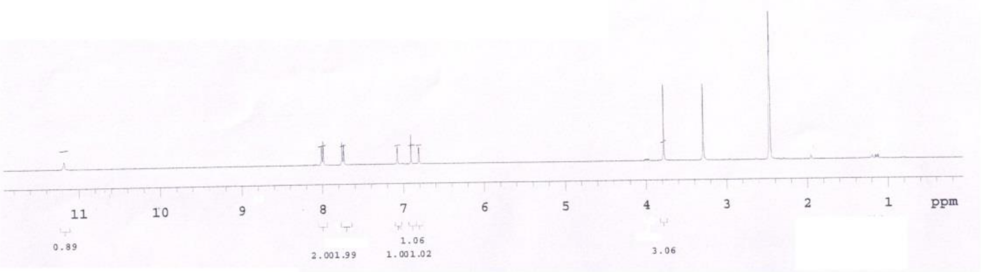

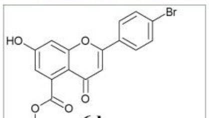

6d

Molecular Formula: $C_{17}H_{11}BrO_5$

Molecular Weight: 375.17

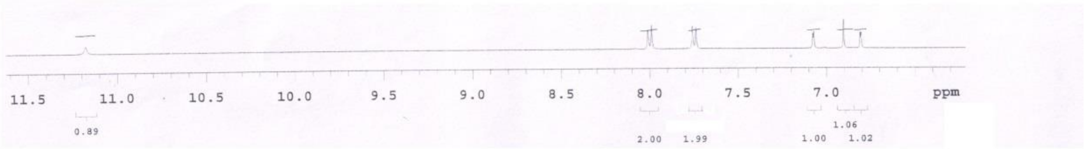

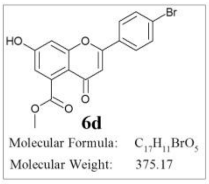
HO
Br
6d
Molecular Formula: C17H11BrO5
Molecular Weight: 375.17

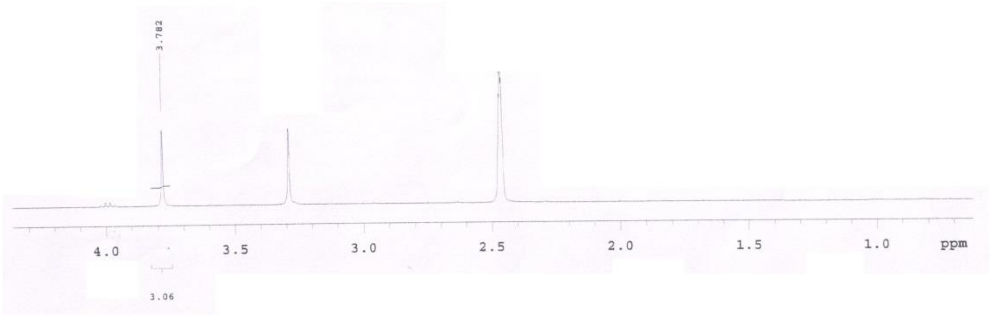
3.782
4.0
3.5
3.0
2.5
2.0
1.5
1.0
ppm
3.06

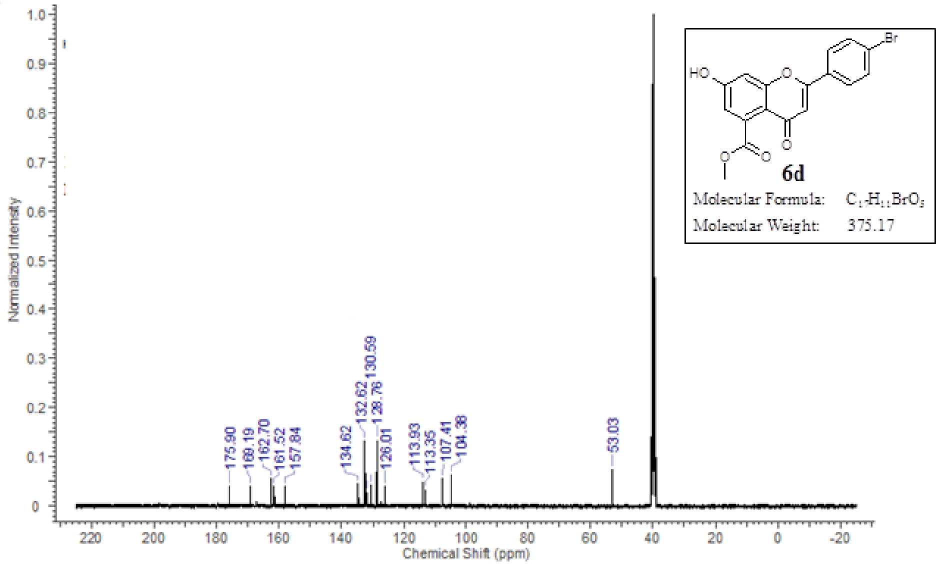
Normalized Intensity
Chemical Shift (ppm)
175.90
169.19
162.70
161.52
157.84
134.62
132.62
130.59
128.76
126.01
113.93
113.35
107.41
104.38
53.03
HO
Br
6d
Molecular Formula: C17H11BrO5
Molecular Weight: 375.17

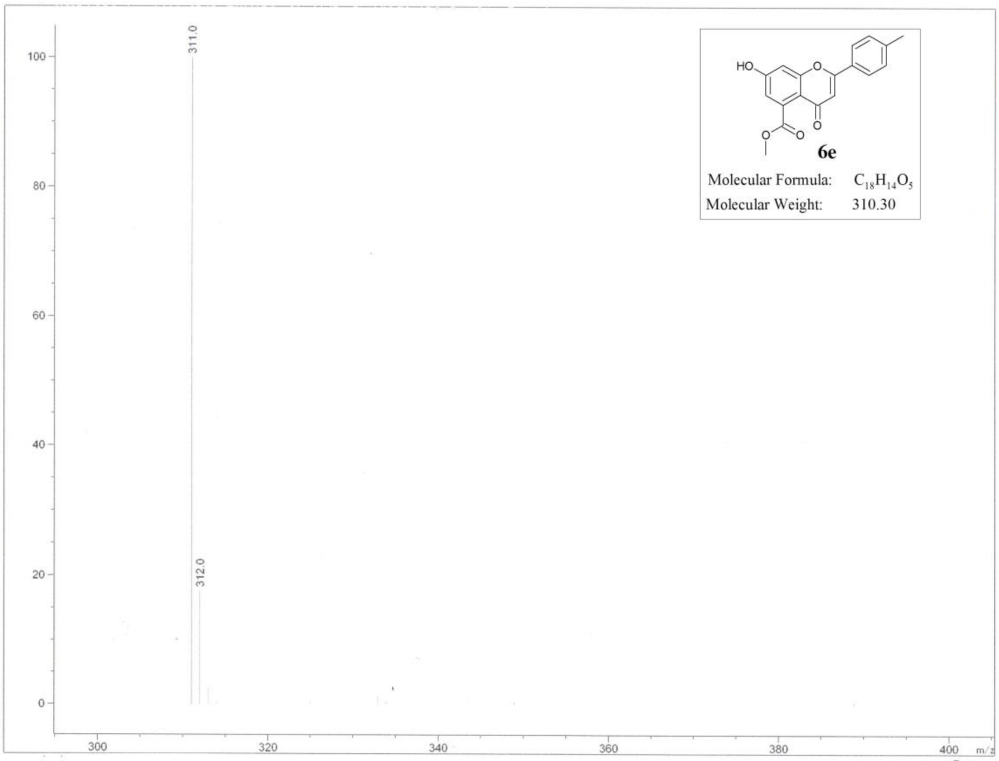
311.0
312.0
100
80
60
40
20
0
300
320
340
360
380
400
m/z
HO
O
O
O
O
6e
Molecular Formula: $C_{18}H_{14}O_5$
Molecular Weight: 310.30

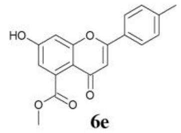

6e

Molecular Formula: $C_{18}H_{14}O_5$

Molecular Weight: 310.30

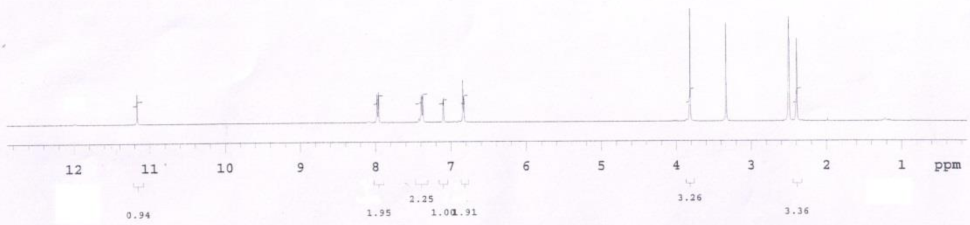

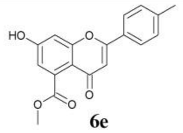

6e

Molecular Formula: $C_{18}H_{14}O_5$

Molecular Weight: 310.30

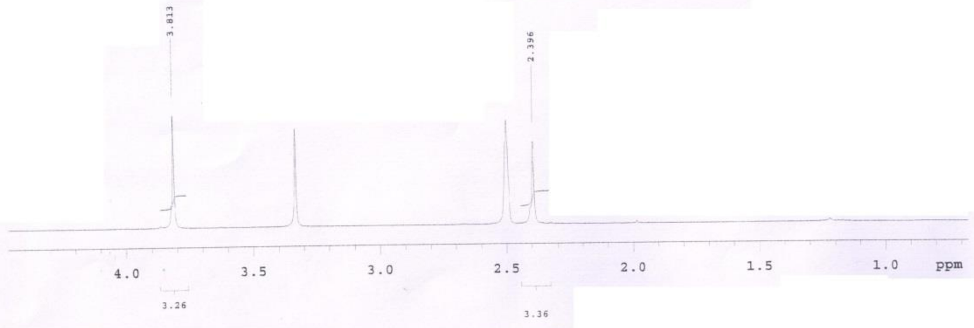

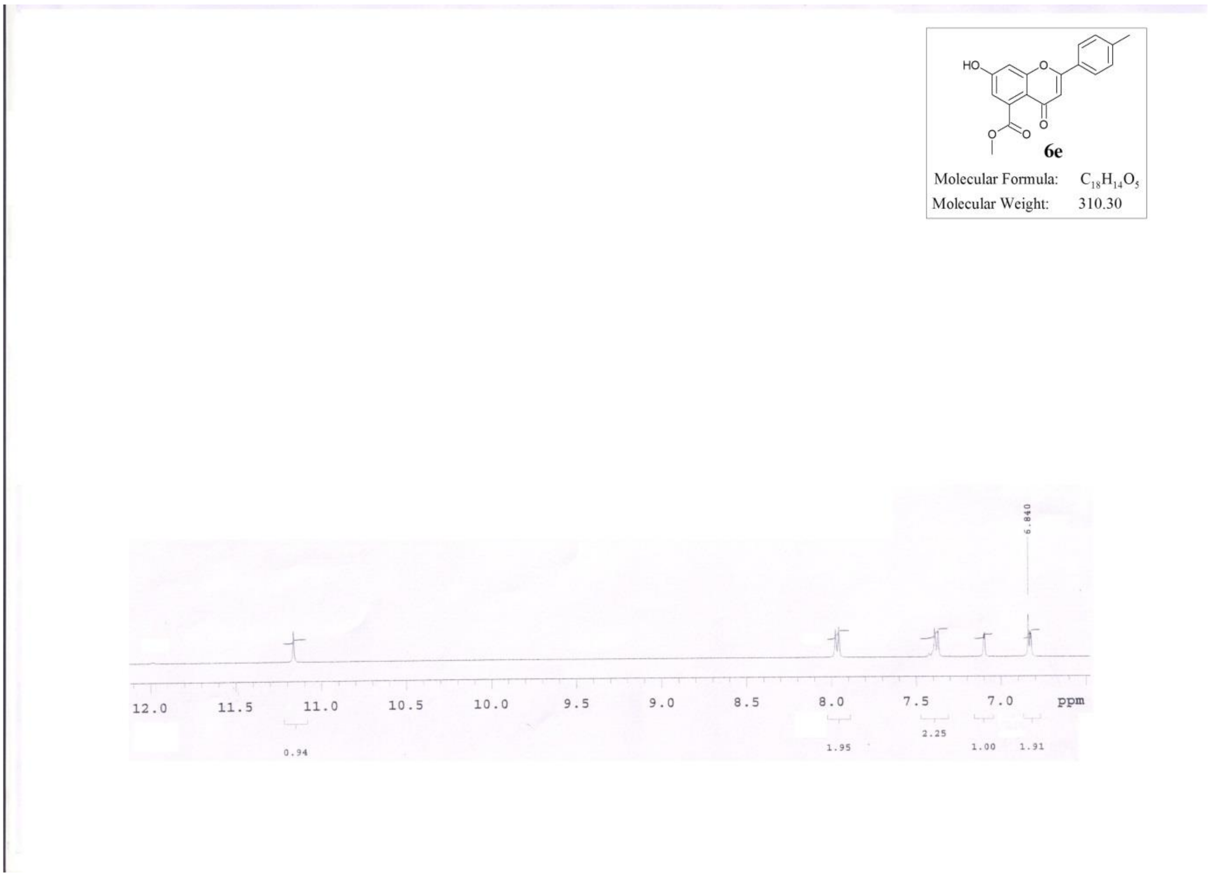
HO
O
O
O
O
6e
Molecular Formula: $C_{18}H_{14}O_5$
Molecular Weight: 310.30
6.840
12.0
11.5
11.0
10.5
10.0
9.5
9.0
8.5
8.0
7.5
7.0
ppm
0.94
1.95
2.25
1.00
1.91

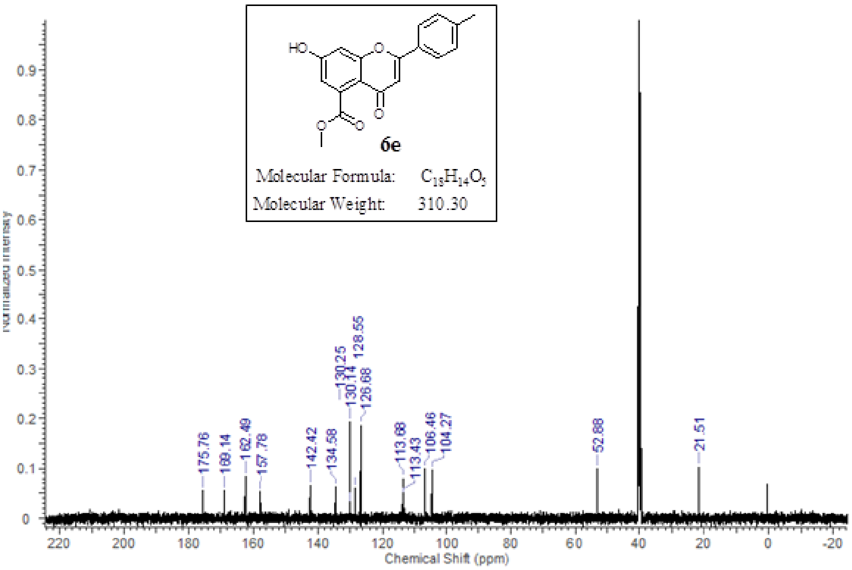
HO
O
O
O
O
6e
Molecular Formula: $C_{18}H_{14}O_5$
Molecular Weight: 310.30
175.76
169.14
162.49
157.78
142.42
134.58
130.25
130.14
128.55
126.68
113.68
113.43
106.46
104.27
52.88
21.51
Chemical Shift (ppm)

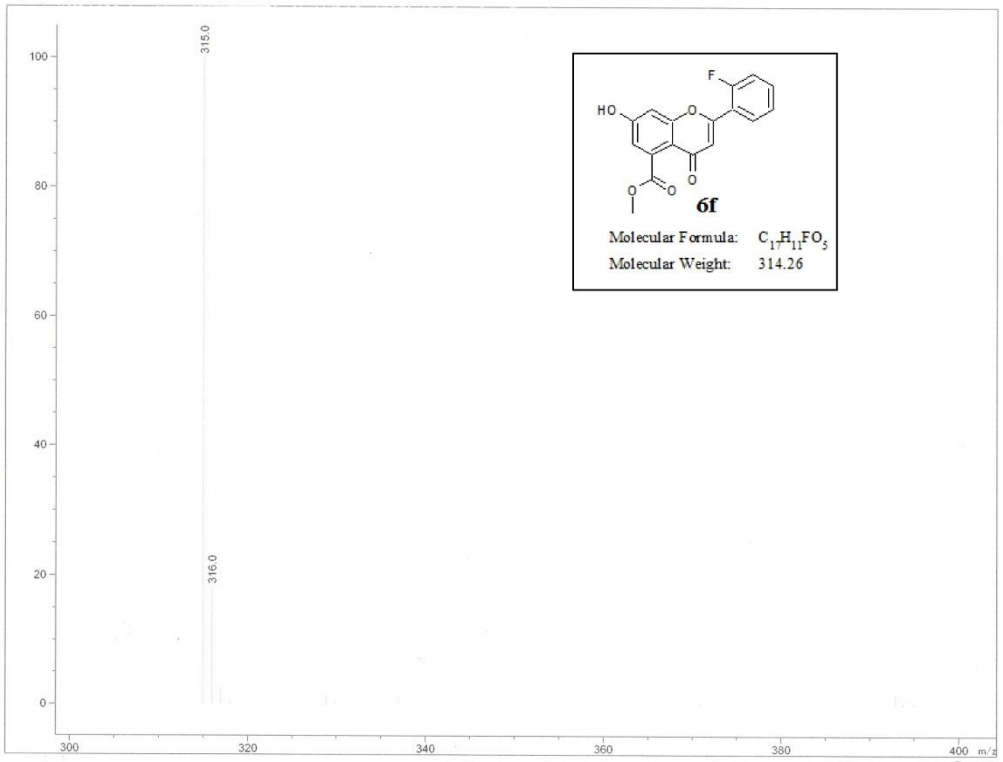
315.0
316.0
HO
F
O
6f
Molecular Formula: $C_{17}H_{11}FO_5$
Molecular Weight: 314.26
100
80
60
40
20
0
300
320
340
360
380
400 m/z

Page 1 of 1

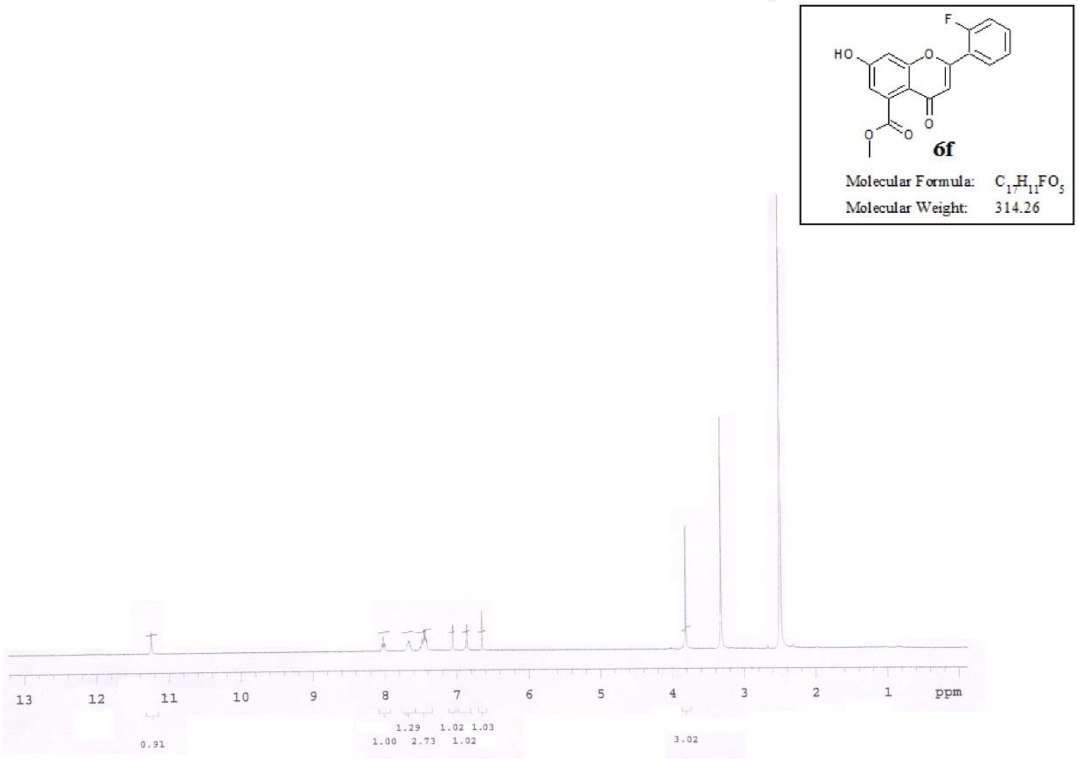
HO
F
O
6f
Molecular Formula: $C_{17}H_{11}FO_5$
Molecular Weight: 314.26
13
12
11
10
9
8
7
6
5
4
3
2
1
ppm
0.91
1.29
1.02 1.03
1.00 2.73 1.02
3.02

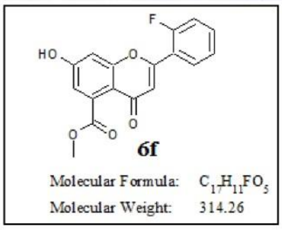

HO
F
6f
Molecular Formula: $C_{17}H_{11}FO_5$
Molecular Weight: 314.26

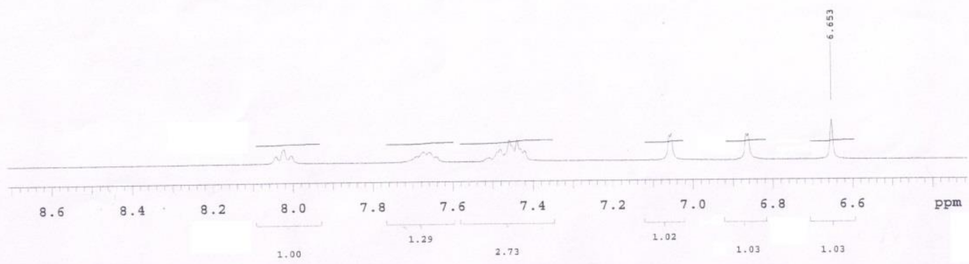

6.653
8.6
8.4
8.2
8.0
7.8
7.6
7.4
7.2
7.0
6.8
6.6
ppm
1.00
1.29
2.73
1.02
1.03
1.03

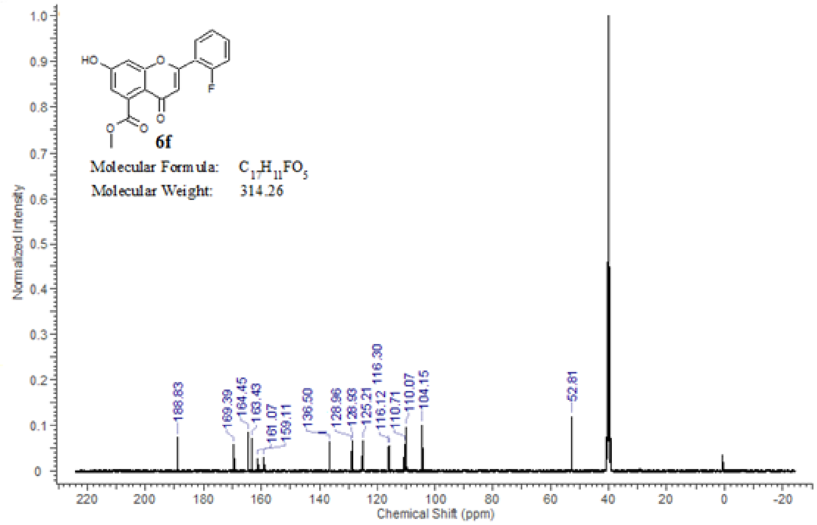

HO
F
6f
Molecular Formula: $C_{17}H_{11}FO_5$
Molecular Weight: 314.26
Normalized Intensity
Chemical Shift (ppm)
188.83
169.39
164.45
163.43
161.07
159.11
136.50
128.96
128.93
125.21
116.30
116.12
110.71
110.07
104.15
52.81
